# Purpurin Alleviates Meniscal Senescence and Osteoarthritis by Blocking the Non‐Metabolic Function of Hexokinase 2

**DOI:** 10.1111/cpr.70294

**Published:** 2026-10-29

**Authors:** Zhencan Lin, Ming Li, Wei Li, Tianfa Xu, Zongrui Jiang, Yanlin Zhong, Aishan He, Xiaoyi Zhao, Dianbo Long, Yan Kang

**Affiliations:** ^1^ Department of Sports Medicine The First Affiliated Hospital, Sun Yat‐sen University Guangzhou Guangdong China; ^2^ Guangdong Provincial Key Laboratory of Orthopedics and Traumatology The First Affiliated Hospital, Sun Yat‐sen University Guangzhou Guangdong China; ^3^ Department of Sports Medicine, Shenzhen Second People's Hospital The First Affiliated Hospital of Shenzhen University Shenzhen Guangdong China; ^4^ Department of Joint Surgery The First Affiliated Hospital, Sun Yat‐sen University Guangzhou Guangdong China

**Keywords:** cellular senescence, hexokinase 2 (HK2), meniscal degeneration, non‐metabolic function, purpurin

## Abstract

Meniscal degeneration, characterised by extracellular matrix (ECM) loss and cellular senescence, plays a critical role in osteoarthritis (OA) progression, yet the underlying mechanisms remain unclear. Although hexokinase 2 (HK2) is recognised as a key glycolytic enzyme, its potential non‐metabolic functions in the meniscus remain unexplored. In this study, HK2 expression was evaluated in human OA and normal meniscus tissues, and its non‐metabolic effects were investigated in primary meniscal cells using overexpression, knockdown, and site‐directed mutagenesis; protein interactions were analysed by immunoprecipitation‐mass spectrometry (IP‐MS) and co‐immunoprecipitation (Co‐IP). HK2 was significantly upregulated in OA meniscal tissues, and overexpression of catalytically inactive HK2 promoted meniscal ECM degradation and cellular senescence in an MYH10‐dependent manner. Mechanistically, HK2 enhanced the MYH10–PRKN interaction, inducing K48‐linked ubiquitination of MYH10 at lysine 410 and its subsequent proteasomal degradation, thereby reducing *COL1A1* mRNA stability. Purpurin, identified through virtual screening, disrupted the HK2–MYH10 interaction in vitro, prevented MYH10 degradation, and attenuated cellular senescence. In the destabilisation of the medial meniscus (DMM) mouse model, intra‐articular injection of adeno‐associated virus expressing catalytically inactive HK2 exacerbated OA progression, whereas purpurin treatment alleviated meniscal degeneration, ECM loss, and OA‐associated pain. Collectively, HK2 promotes meniscal senescence and OA progression through a non‐metabolic, MYH10‐dependent mechanism, and purpurin represents a promising lead compound for disease‐modifying osteoarthritis drug (DMOAD) development.

## Graphical abstract

HK2 is upregulated in osteoarthritis and accelerates disease progression via a glycolysis‐independent non‐metabolic mechanism. It recruits PRKN to degrade MYH10, reducing *COL1A1* mRNA stability and inducing senescence. Purpurin disrupts this pathogenic pathway with therapeutic potential.

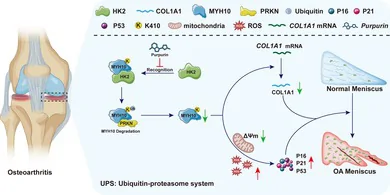

## 1 Introduction

Osteoarthritis (OA) is a widespread and debilitating disease affecting nearly 600 million individuals worldwide [1], thus posing a major global public health burden [2]. Meniscal injuries are frequently associated with an increased risk of developing OA [3, 4, 5, 6]. The meniscus is a highly organised extracellular matrix (ECM)‐rich tissue located between the femur and tibia, primarily composed of Type I and Type II collagen, with proteoglycans distributed among the collagen fibres. Type I collagen accounts for more than 90% of the dry weight of the meniscal ECM [7, 8], contributing to proper stress distribution and maintenance of normal knee joint function [9]. Meniscal degeneration predominantly occurs in the avascular white zone, where the loss of Type I collagen is particularly pronounced [10, 11, 12]. This loss impairs the regenerative capacity of the meniscus following injury. Therefore, degradation of Type I collagen is considered a key pathological factor contributing to OA development and progression.

In addition, cellular senescence is another critical pathological process in OA characterised by irreversible cell‐cycle arrest, functional decline, and increased secretion of senescence‐associated secretory phenotype (SASP) factors. Enhanced SASP secretion promotes ECM degradation and exacerbates tissue damage [13, 14, 15]. Moreover, mitochondrial dysfunction in senescent cells drives excessive reactive oxygen species (ROS) production, leading to oxidative stress and DNA damage, which further exacerbate cellular injury and functional deterioration [16, 17, 18]. In contrast, clearance of senescent cells can establish a pro‐regenerative microenvironment [19, 20]. OA is characterised by degenerative changes in multiple joint tissues, including the synovium, cartilage, and meniscus [17]. Studies have implicated synovial [21] and chondrocyte [22] senescence in OA progression, and reversal of senescence mitigates disease progression [22]. However, the role of meniscal senescence in OA remains poorly understood.

As the first rate‐limiting enzyme in the glycolytic pathway, HK2 plays a critical role in maintaining energy metabolism and adaptation to hypoxia [23]. The meniscus is a relatively hypoxic tissue, and meniscal cells primarily rely on glycolysis for energy production [24], making HK2 essential for maintaining meniscal homeostasis. Although the metabolic functions of HK2 have been extensively characterised, growing evidence suggests that it also exerts non‐glycolytic regulatory roles beyond glucose phosphorylation. In acute myeloid leukaemia and normal haematopoiesis, nuclear‐localised HK2 regulates stem/progenitor cell function and differentiation [25]. In prostate cancer, loss of HK2 SUMOylation promotes mitochondrial binding of HK2, thereby enhancing tumour proliferation and chemoresistance [26]. HK2 also functions as a protein kinase by phosphorylating sphingomyelinase 1, thereby exacerbating cerebral injury following arterial occlusion [27]. However, the non‐metabolic role of HK2 in regulating meniscal function remains unclear.

Purpurin is a natural anthraquinone derived from *Rubia cordifolia*. Studies have demonstrated its potent antioxidant and anti‐inflammatory activities, including free radical scavenging, reduction of intracellular ROS levels, inhibition of NLRP3 inflammasome assembly, and downregulation of the pro‐inflammatory cytokine interleukin‐1β [28, 29]. Although these biological activities have been validated in various in vitro and in vivo models, the potential protective effects of purpurin against meniscal senescence or OA progression remain unknown.

In the present study, we found that HK2 was significantly upregulated in OA meniscal tissues, where it interacted with non‐muscle myosin heavy chain IIB (MYH10) and promoted the interaction between MYH10 and PRKN. This interaction induced MYH10 ubiquitination and degradation, destabilised *COL1A1* mRNA, enhanced cellular senescence, and reduced the ECM‐producing phenotype. Importantly, purpurin disrupted the HK2–MYH10 interaction, thereby preventing MYH10 degradation, restoring collagen expression, and attenuating meniscal senescence.

Collectively, this study reveals a non‐canonical, activity‐independent role of HK2 in OA pathogenesis and highlights purpurin‐mediated disruption of the HK2–MYH10 interaction as a potential therapeutic strategy for DMOAD development.

## 2 Materials and Methods

### 2.1 Human Sample Collection, Isolation, and Culture

Human OA menisci were obtained from patients undergoing total knee arthroplasty. Normal menisci (control) were obtained from patients with no history of arthritis who underwent amputation for severe trauma. Before collecting the meniscus samples, informed consent was obtained from all participating patients. Detailed demographic and clinical baseline characteristics of the patients are summarised in Table S1. Histopathological assessment of human menisci was performed using the semi‐quantitative scoring system established by Pauli et al. [30].

The harvested meniscus samples were sliced into smaller fragments, followed by sequential enzymatic digestion. This process involved the use of 4 mg/mL protease (P5147, Sigma‐Aldrich) for 90 min, followed by 0.25 mg/mL collagenase P (11,213,873,001, Roche, Mannheim, Germany) for 12 h. Primary meniscal cells were placed into 75 cm^2^ culture flasks and cultured using DMEM/F‐12 medium (Gibco Life Technologies), supplemented with 10% fetal bovine serum (FBS, Gibco Life Technologies) and 1% penicillin–streptomycin (Gibco Life Technologies), under a 5% CO_2_ environment at 37°C.

### 2.2 TBHP‐Induced Senescence‐Like Model

Meniscal cells were treated with 25 or 50 μM Tert‐Butyl Hydroperoxide (TBHP) in complete DMEM/F‐12 medium for 24 h at 37°C in 5% CO_2_ to induce oxidative stress‐associated senescence‐like changes.

### 2.3 RNA Extraction, Reverse Transcription, and RT‐qPCR

Total RNA was extracted from meniscal cells using the SteadyPure RNA Extraction Kit (AG21024, Accurate Biology) according to the manufacturer's instructions. Reverse transcription was performed using the EvoM‐MLV RT Premix (AG11706, Accurate Biology). Quantitative real‐time PCR (qPCR) was carried out using the SYBR Green ProTaq HS Premix (AG11701, Accurate Biology) on an ABI ViiA 7 Real‐Time PCR System (Applied Biosystems, Foster City, CA, USA). Primer sequences used for qPCR are listed in Table S2. Gene expression levels were determined using the 2^−ΔΔCt^ method.

### 2.4 RNA Immunoprecipitation (RIP)

RIP was performed using the PureBinding RNA Immunoprecipitation Kit (Geneseed, P0101) according to the manufacturer's instructions. Magnetic beads were incubated with 5 μg of anti‐MYH10 antibody (Proteintech, 19673‐1‐AP) or normal rabbit IgG (Abcam, ab172730). The antibody‐coated beads were then added to meniscal cell lysates and incubated at 4°C for 12 h. Subsequently, proteinase K digestion buffer was added to digest the immunoprecipitated complexes at 55°C for 45 min. RNA was extracted with phenol:chloroform:isoamyl alcohol (125:24:1) and analysed by RT‐qPCR. Normal rabbit IgG served as a negative control to exclude nonspecific binding.

### 2.5 Western Blot

Meniscal cells were lysed on ice in RIPA lysis buffer (Beyotime) supplemented with PMSF, and the supernatant was collected after centrifugation. The protein concentration was assessed using a BCA Protein Assay Kit (Thermo Fisher). Total protein was then separated via SDS‐PAGE and transferred to PVDF membranes (Millipore) for further analysis. After blocking, the membranes were incubated overnight at 4°C with primary antibodies targeting COL1A1, MMP3, HK2, MYH10, PRKN, LC3, P62, ubiquitin, K48‐Ub, K63‐Ub, HA, Flag, and ACTB. Information regarding antibody sources, catalogue numbers, and dilutions is available in Table S3. Following this incubation, HRP‐conjugated secondary antibodies were added at room temperature for 1 h. Finally, protein bands were visualised using an ECL solution (Millipore) and detected with an automatic chemiluminescence imaging analysis system (Bio‐Rad). Semi‐quantitative analysis was conducted employing ImageJ software (version 1.51a).

### 2.6 SA‐β‐Gal Staining

The staining for senescence‐associated β‐galactosidase (SA‐β‐Gal) was performed utilising the Senescence β‐Galactosidase Staining Kit provided by Beyotime (catalogue number C0602), with strict adherence to the guidelines specified by the manufacturer. Initially, the cells were fixed using the β‐galactosidase staining fixative for 15 min. Following this fixation process, the cells were incubated in the staining solution overnight at 37°C. After the staining was completed, SA‐β‐Gal‐positive cells were carefully counted in three randomly selected fields per well.

### 2.7 Tissue Immunofluorescence (IF)

To perform tissue immunofluorescence (IF), 6‐μm‐thick paraffin‐embedded mouse meniscus sections were deparaffinized with xylene and a series of graded ethanol solutions before being rehydrated. Antigen retrieval was performed using a citric acid buffer at pH 6.0. Following this, the sections were treated with 3% H_2_O_2_ to block endogenous peroxidase and then blocked with goat serum. They were incubated with primary antibodies overnight at 4°C, then treated with secondary antibodies for 1 h at room temperature, concluding with DAPI staining. The samples were examined and analysed using fluorescence or confocal laser scanning microscopy.

### 2.8 Cell Transfection

Meniscal cells were seeded in six‐well plates and cultured until reaching approximately 80% confluence. For overexpression, plasmids encoding HK2, MYH10, or PRKN were constructed using the pcDNA3.1 vector. For knockdown, short hairpin RNA (shRNA) plasmids targeting HK2, MYH10, or PRKN were constructed using the pLKO.1 vector. Transfection was performed using Lipofectamine 3000 (Invitrogen, USA) according to the manufacturer's instructions. Cells were harvested for RT‐qPCR analysis 48 h post‐transfection and for western blot 72 h post‐transfection.

### 2.9 Transwell Co‐Culture Assay

Meniscal cells were seeded in 24‐mm Transwell inserts with 0.4‐μm pores and transfected with HK2‐overexpressing or empty vector plasmids. At 6–8 h post‐transfection, the transfection medium was removed, and the inserts were gently washed with PBS and transferred to six‐well plates containing normal meniscal cells in the lower chambers. Fresh complete DMEM/F‐12 medium was added, and donor and recipient cells were co‐cultured without direct cell‐to‐cell contact for 72 h. The inserts were then removed, and recipient cells in the lower chambers were harvested for western blot analysis.

### 2.10 NAC Treatment

To inhibit reactive oxygen species (ROS), the ROS scavenger N‐acetyl‐L‐cysteine (NAC; A9165, Sigma‐Aldrich) was dissolved in PBS and added to meniscal cells at a final concentration of 5 mM at the time of transfection or stimulation, and replenished every 24 h for 72 h. Control cells received an equal volume of vehicle. Cells were harvested for protein analyses as described above.

### 2.11 mRNA Stability Assay

To determine mRNA half‐life, meniscal cells were treated with actinomycin D (5 μg/mL; HY‐17559, MedChemExpress) to block transcription, and total RNA was harvested at 0, 4, and 8 h. *COL1A1* mRNA levels at each time point were quantified by RT‐qPCR and normalised to ACTB. Decay data were fitted to a one‐phase exponential decay model (plateau = 0) and the half‐life was calculated as t_1/2_ = ln(2)/K. Group differences were compared by the extra sum‐of‐squares F test; *p* < 0.05 was considered significant.

### 2.12 Mitochondrial Membrane Potential (JC‐1) Detection

Staining with JC‐1 was carried out utilising an enhanced mitochondrial membrane potential assay kit (C2003S, Beyotime), following the guidelines provided by the manufacturer. To prepare the JC‐1 staining working solution, JC‐1 (200×) was diluted into the staining buffer at a ratio of 5 μL of JC‐1 (200×) per 1 mL of buffer. For the staining process, 1 mL of the working solution was added to the cells and incubated at 37°C for 20 min. Following this, the supernatant was removed, and the cells were rinsed twice with the JC‐1 staining buffer. Subsequently, each well received 1 mL of 1× Hoechst 33342 solution and was incubated for 10 min at room temperature in the dark. After discarding the Hoechst 33342 solution, the cells underwent three washes with 1 mL of PBS per well, and 2 mL of serum‐free culture medium was added back. Ultimately, the cells were examined through flow cytometry.

### 2.13 Reactive Oxygen Species (ROS) Staining

Meniscal cells were stained with diluted 2′,7′‐dichlorofluorescin diacetate (DCFH‐DA) and incubated at 37°C for 20 min according to the manufacturer's protocol (S0033M, Beyotime). Subsequently, the meniscal cells were washed twice with phosphate‐buffered saline (PBS, Servicebio, Wuhan, China) to eliminate excess DCFH‐DA. Finally, the cells were observed and analysed using a fluorescence microscope.

### 2.14 Hexokinase (HK) Activity Analysis

The activity of hexokinase (HK) in meniscal cells was evaluated using the hexokinase activity assay kit (Abcam, ab136957) in accordance with the manufacturer's guidelines. Meniscal cells were swiftly homogenized in HK assay buffer while kept on ice for 10 min. After centrifugation, the supernatant was collected for colorimetric measurement at OD 450 nm. The quantification of hexokinase activity was performed by creating an NADH standard curve reflecting the OD values, and the levels of cellular NADH were normalised to the protein concentration of the samples.

### 2.15 Immunoprecipitation‐Mass Spectrometry (IP‐MS)

To identify the target protein that binds to HK2, primary meniscal cells were lysed on ice for 30 min using IP Lysis/Wash Buffer (IK‐1004, Biolinkedin). The supernatant was collected after centrifugation. Anti‐HK2 antibodies (22029‐1‐AP, Proteintech) were added to form immune complexes, and subsequent experiments were conducted according to the manufacturer's protocol to obtain the enriched target proteins.

The silver‐stained bands were washed three times with ddH_2_O and then treated with 150 μL of decolorization solution, followed by four rinses with water. The gel strips were sequentially washed twice each with 300 μL of 25 mM ammonium bicarbonate, 50% acetonitrile (ACN), and 100% ACN, and then dehydrated until the gel pieces turned white. Next, 50 μL of 10 mM DTT solution was added, and the mixture was incubated in a 56°C water bath for 30 min. After cooling to room temperature, an equal volume of 50 mM IAA solution was added, and the mixture was alkylated in the dark for 15 min. The gel strips were washed again twice each with 300 μL of 25 mM ammonium bicarbonate, 50% ACN, and 100% ACN, followed by dehydration until the gel pieces turned white. Then, 15–20 μL of proteomics‐grade trypsin (0.01 μg/μL) was added, and the gel pieces were allowed to swell on ice until transparent. After adding 30–40 μL of 50 mM NH₄HCO₃ solution containing 10% ACN, the digestion was carried out at 37°C overnight. The supernatant was transferred to a new EP tube, and 100 μL of extraction solution (67% ACN with 2% formic acid) was added to the remaining gel pieces. After incubation at 37°C for 30 min and 15 min of sonication, the supernatants were combined and dried for subsequent mass spectrometry analysis.

The enzymatically digested peptide samples were reconstituted in the Nano‐LC mobile phase A, consisting of 0.1% formic acid in water, and subsequently loaded onto a nanoViper C18 trap column for online LC–MS analysis. Liquid chromatography was performed using an Easy nLC 1200 system, and the samples were separated on an analytical C18 reverse‐phase column. The gradient was established to increase mobile phase B, which consisted of 80% acetonitrile (ACN) and 0.1% formic acid, from 5% to 38% over 60 min. Mass spectrometry was conducted using a Thermo Fisher Q Exactive system coupled with a nano‐flex ion source. The raw data files were then analysed using PEAKS Studio 8.5.

### 2.16 Molecular Docking

To characterise both protein–protein and drug–protein interactions, two complementary docking approaches were employed. For protein–protein docking, the structures of HK2–MYH10 and MYH10–PRKN were submitted to the HDOCK online server (http://hdock.phys.hust.edu.cn/), which uses a fast Fourier transform algorithm to explore potential binding modes and a knowledge‐based scoring function to evaluate the sampled modes; the complex with the most favourable (most negative) docking score was selected and its interactions were analysed with PyMOL 3.1. For drug–target protein docking, the drug and key target protein structures were retrieved from the PubChem (https://pubchem.ncbi.nlm.nih.gov) and PDB (https://www.rcsb.org) databases, respectively. Water molecules were removed from the protein structures using PyMOL 3.1, and both protein and ligand were prepared with AutoDockTools 1.5.7. Molecular docking was carried out with AutoDock Vina 1.1.2, after which chemical bond interactions were analysed with LigPlot+ and visualisations were generated with PyMOL 3.1.

### 2.17 Virtual Screening

Based on the human HK2 crystal structure (PDB ID: 2NZT), the binding pocket was centered on residues Y865, K866, and Q892 at the putative MYH10‐interacting surface. The protein was prepared with PyMOL 3.1: water molecules were removed, missing atoms and side chains were added, and the backbone geometry was optimised. A library of 3219 natural products from the Traditional Chinese Medicine Active Compound Library (HY‐L065, MCE) was used. All ligands were energy‐minimised with the MM2 force field (1000 iterations, convergence threshold 0.001 kcal·mol^−1^·Å^−1^). Using AutoDockTools 1.5.7, Gasteiger charges were added to the protein, and ligand atom types and rotatable bonds were assigned. Molecular docking was performed with Vina‐GPU 2.1, generating 9 poses per ligand. Compounds were ranked by binding energy, and the top five candidates (all with binding energies more favourable than −5.0 kcal/mol) were selected; their structures and docking scores are listed in Figure S5A. Hydrogen bonds and hydrophobic interactions were analysed with LigPlot+, and binding modes were visualised with PyMOL 3.1. The selected complexes were further validated by molecular dynamics simulations.

### 2.18 Molecular Dynamics Simulation

To obtain atomic‐level insights into the dynamic binding mode and conformational changes of the complex under physiological conditions, molecular dynamics simulations were performed using GROMACS 2026.1 with the CHARMM36 force field. The complex was solvated in a cubic TIP3P water box, leaving a 10 Å buffer from the protein surface. Energy minimization was carried out using the steepest descent algorithm until the maximum force fell below 1000 kJ·mol^−1^·nm^−1^. The system was then equilibrated in two stages: a 100 ps NVT simulation at 310 K with the V‐rescale thermostat, followed by a 100 ps NPT simulation at 1 bar with the Berendsen barostat. Finally, a 100 ns unrestrained production run was conducted in the NPT ensemble with a 2 fs time step, and coordinates were saved every 10 ps, yielding 10,000 snapshots for subsequent analysis.

### 2.19 Cell Viability Assay

The Cell Counting Kit‐8 (CCK‐8) (C0038, Beyotime) was employed to assess cell viability. Meniscal cells were plated in 96‐well plates, with a total volume of 100 μL of complete culture medium allocated to each well. Then, 10 μL of the CCK‐8 reagent was introduced into each well, while blank wells contained only culture medium and CCK‐8 (without cells). After an additional hour of incubation at 37°C, absorbance readings were taken at 450 nm utilising a Synergy H1 microplate reader (BioTek Instruments, USA).

### 2.20 Destabilisation of the Medial Meniscus (DMM) Mouse Model of OA

This study employed 12‐week‐old male wild‐type C57BL/6 mice. Mice were anaesthetised by intraperitoneal injection of 1% sodium pentobarbital at 50 mg/kg. For mice assigned to the DMM groups, DMM surgery was performed on the left knee. A medial parapatellar skin incision was made, followed by exposure of the knee joint under a binocular microscope. After identification of the medial meniscus, the medial meniscotibial ligament was transected to destabilise the medial meniscus. Mice in the Sham group underwent the same surgical exposure without transection of the medial meniscotibial ligament. The joint capsule and skin were then closed layer by layer using 5–0 absorbable sutures, and the surgical site was disinfected. After recovery from anaesthesia, mice were returned to their cages after confirming normal movement of the lower limbs. Tissue specimens were fixed in 4% paraformaldehyde (PFA) for 2 days, then embedded in paraffin after decalcification with ethylenediaminetetraacetic acid (EDTA) for a week. The paraffin blocks were sliced into 6 μm thick sagittal sections. Staining techniques using SO/FG and haematoxylin and eosin (H&E) were performed, with joint pathology quantified through OARSI scoring [31]. Histopathological evaluation of the medial meniscus was conducted on SO/FG‐stained sections using the semi‐quantitative scoring system established by Kwok et al. [32].

### 2.21 Intra‐Articular Injection of AAV9 Overexpressing HK2/Mut‐HK2 in the DMM Mouse Model

Mice received intra‐articular injections of 10 μL rAAV9, rAAV9‐HK2, or rAAV9‐mut‐HK2 once weekly for four consecutive weeks. Knee joints were harvested 8 weeks after DMM surgery for subsequent analysis. AAV9‐mediated transgene expression was confirmed by GFP fluorescence in the meniscus, articular cartilage, and synovium (Figure S3H).

### 2.22 Intra‐Articular Injection of Purpurin in the DMM Mouse Model

Mice received intra‐articular injections of purpurin beginning 1 week after DMM surgery. Purpurin was first dissolved in DMSO to prepare a 50 mM stock solution and then diluted with sterile normal saline to a final concentration of 100 μM, with a final DMSO concentration of 0.2%. A total volume of 10 μL purpurin solution (100 μM) was injected into the operated knee joint once weekly for 7 consecutive weeks. Control mice received an equal volume of vehicle consisting of normal saline containing 0.2% DMSO according to the same schedule. Knee joints were harvested 8 weeks after DMM surgery for subsequent analyses.

### 2.23 Vocalization Test

To evaluate hindlimb joint pain, the mice were tested at 8 weeks after DMM surgery. While the mice were awake and unrestrained, each knee joint was subjected to forced flexion and extension (10 repetitions at 5‐s intervals). Each manipulation was assigned a binary score of 0 (no vocalisation) or 1 (vocalisation present). The cumulative number of vocalisations detected was recorded as the vocalisation score [33].

### 2.24 Micro‐CT Analysis

After fixation in 4% paraformaldehyde (PFA) for 2 days and before decalcification, all knee specimens were analysed using the Bruker SkyScan‐2211 nano‐computed tomography (CT) device (Bruker micro‐CT, Kontich, Belgium). These micro‐CT imaging processes were carried out following the manufacturer's specifications. The scanner acquired projection images over a 180 degrees rotation, operating at a voltage of 90 kVp and a current of 450 μA. To reduce beam hardening artefacts, a 0.5‐mm aluminium (Al) filter was utilised. The reconstruction of three‐dimensional images was performed with Mimics Medical 20.0 software.

### 2.25 Statistical Analysis

Data analysis was carried out using IBM SPSS Statistics version 23.0. To examine the distribution of the dataset, the Shapiro–Wilk test was employed, while the Levene test assessed the equality of variances. An unpaired two‐tailed Student's *t*‐test was used for comparing two groups of normally distributed data with equal variances. In situations where variances were unequal, a Welch *t*‐test was utilised, and for datasets that did not follow a normal distribution, the Mann–Whitney *U* test was applied. For comparisons involving multiple groups, a one‐way ANOVA was conducted for data that were normally distributed, whereas a Kruskal–Wallis test was performed for non‐normally distributed data, followed by Dunn's test with Bonferroni correction for multiple comparisons. Statistical significance was established at *p* < 0.05. In all experiments, “*n*” denotes the number of independent biological replicates (independent donors for primary meniscal cells, independent mice for animal experiments, and independent patient samples for human tissue analyses). Technical replicates, when performed, were not treated as independent observations. All histological scoring and image quantifications were performed independently by two investigators blinded to group allocation and clinical information; any discrepancies were resolved by discussion between the two investigators until consensus was reached.

## 3 Results

### 3.1 Significant Upregulation of HK2 in the OA Meniscus

Knee OA is frequently associated with degenerative changes in the meniscus (Figure 1A). Senescence‐associated beta‐galactosidase (SA‐β‐Gal) staining revealed that the OA meniscus (OM) contained a higher proportion of senescent cells (Figure 1B; Figure S1A). To identify potential therapeutic targets for OA, we analysed an RNA‐seq dataset (GSE335572) comparing normal meniscus (NM) and OM samples. Differential gene expression analysis demonstrated that the glycolysis‐related gene *HK2* was significantly upregulated in OA meniscal tissues (Figure 1C).

**FIGURE 1:**
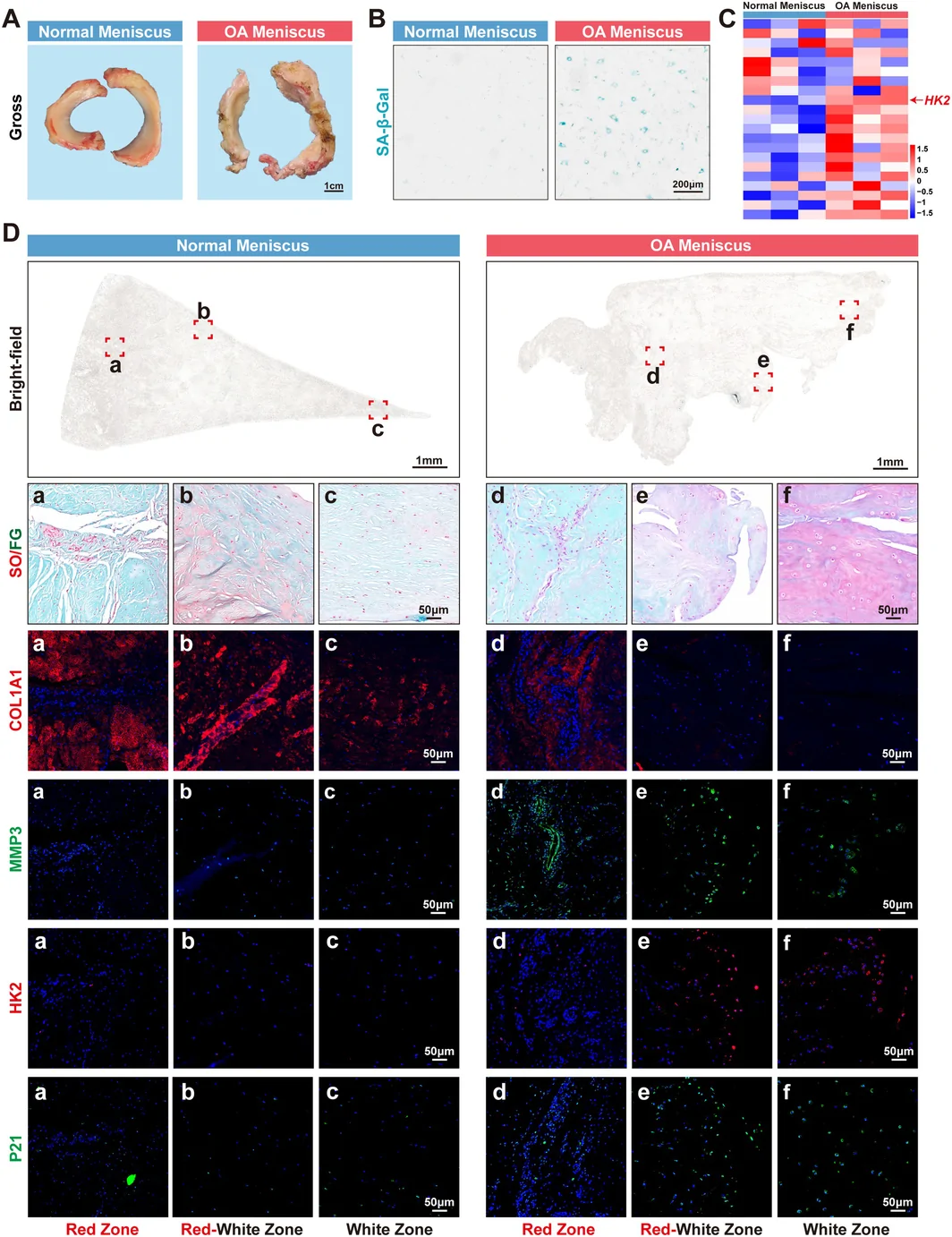
Significant upregulation of HK2 in the OA meniscus. (A) Gross morphology of normal and OA meniscus. (B) SA‐β‐Gal staining of normal and OA meniscal cells (*n* = 3). Scale bars = 200 μm. (C) *HK2* mRNA expression in normal and OA meniscus by RNA‐seq (*n* = 3). (D) SO/FG staining and immunofluorescence for COL1A1, MMP3, P21, and HK2 in normal and OA meniscus. Scale bars = 1 mm (Top) and 50 μm (Bottom).

Western blot analysis of clinically obtained NM and OM tissue samples revealed a significant decrease in Type I collagen (COL1A1; an anabolic marker) expression in OM, whereas matrix metalloproteinase 3 (MMP3; a catabolic marker), HK2 and senescence‐associated markers (P16, P21, and P53) were significantly upregulated (Figure S1B,C). Consistently, transcriptional analysis also showed elevated *HK2* mRNA expression in OM (Figure S1D). Furthermore, HK2 expression was significantly increased at both the mRNA and protein levels in normal meniscal cells treated with tert‐butyl hydroperoxide (TBHP) to induce oxidative stress‐associated senescence‐like changes (Figure S1E,F).

Histological evaluation using Safranin O/Fast Green (SO/FG) staining revealed more severe degeneration in the OA meniscal tissues. The OM exhibited a disrupted and disorganised structure with increased cellular density, resulting in a higher histopathological score (Figure 1D; Figure S1G). Immunofluorescence (IF) analysis demonstrated a significant decrease in COL1A1 expression and significant increases in MMP3 and P21 expression in OM (Figure 1D; Figure S1H–J). In addition, IF staining confirmed marked upregulation of HK2 in OM (Figure 1D; Figure S1K). Consistent with these findings, HK2 expression was significantly elevated in the menisci of both DMM‐induced OA mice and naturally aged mice (18 months old) (Figure S1L).

### 3.2 HK2 Overexpression Exacerbates Meniscal Collagen Loss and Induces Cellular Senescence

To investigate the regulatory role of HK2 in the meniscus, HK2 knockdown and overexpression experiments were performed in cells isolated from normal meniscal tissues. Compared with the control group, HK2 overexpression reduced COL1A1 expression and increased MMP3 and senescence‐associated markers (P16, P21, and P53) expression. In addition, HK2 overexpression significantly increased the number of senescent meniscal cells (Figure 2A,B,E,F). In contrast, HK2 knockdown produced the opposite effects, promoting anabolic activity and suppressing cellular senescence (Figure 2A,B,E,F). Studies have suggested a strong association between HK2 expression and mitochondrial function [26, 34]. In the present study, HK2 overexpression significantly reduced mitochondrial membrane potential in meniscal cells (Figure 2C,D), accompanied by increased P21 expression and ROS generation (Figure 2G; Figure S2A,B). Previous reports have also shown that HK2 can regulate cellular functions through non‐metabolic mechanisms [25, 26, 27, 35]. To determine whether HK2‐induced oxidative stress and cellular senescence were dependent on its metabolic or non‐metabolic functions, we measured total hexokinase activity in NM and OM. The results showed no significant difference in hexokinase activity between NM and OM (Figure 2H). Similarly, TBHP treatment did not alter hexokinase activity in normal meniscal cells (Figure 2I). These findings suggest that although *HK2* mRNA and protein expression were elevated in OM, its enzymatic activity was not correspondingly increased.

**FIGURE 2:**
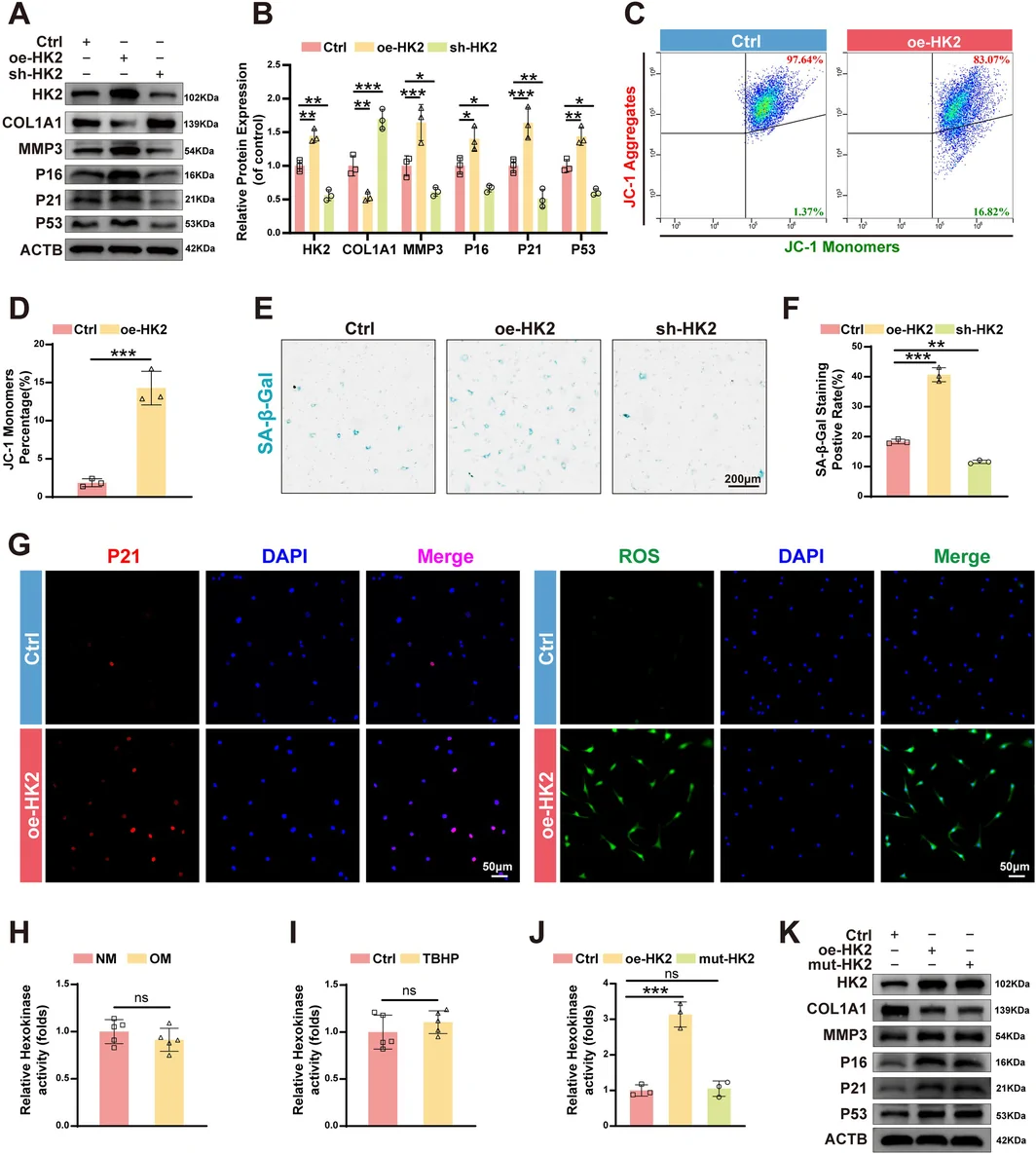
HK2 overexpression exacerbates meniscal collagen loss and induces cellular senescence. (A, B) Western blot and quantification of HK2, COL1A1, MMP3, P16, P21, P53, and ACTB after HK2 overexpression (oe‐HK2) or knockdown (sh‐HK2) (*n* = 3). (C, D) Mitochondrial membrane potential by JC‐1 staining and flow cytometry (*n* = 3). (E, F) SA‐β‐Gal staining and quantification after HK2 overexpression or knockdown (*n* = 3). Scale bars = 200 μm. (G) Immunofluorescence for P21 and ROS after HK2 overexpression (*n* = 3). Scale bars = 50 μm. (H) Hexokinase activity in normal and OA meniscus (*n* = 5). (I) Hexokinase activity in cells treated with TBHP (50 μM, 24 h) or control (*n* = 5). (J) Hexokinase activity in oe‐HK2 or catalytically inactive mut‐HK2 cells (*n* = 3). (K) Western blot of HK2, COL1A1, MMP3, P16, P21, P53, and ACTB in oe‐HK2 and mut‐HK2 cells (*n* = 3). NM, normal meniscus; OM, OA meniscus. Data are presented as mean ± SD, *p < 0.05, **p < 0.01, ***p < 0.001, ns, not significant.

To further investigate whether these effects depended on the metabolic activity of HK2, we constructed a plasmid carrying double‐site mutations (D209A and D657A) designed to abolish HK2 catalytic activity (Figure S2C) [25, 36]. Consistent with this design, mut‐HK2 overexpression did not increase total cellular hexokinase activity above control levels, whereas wild‐type HK2 overexpression significantly increased hexokinase activity (Figure 2J). Unexpectedly, mut‐HK2 retained the ability to promote meniscal catabolism, suppress anabolism, and enhance cellular senescence (Figure 2K; Figure S2D), indicating that these effects were independent of HK2 metabolic activity. Together with the unchanged hexokinase activity in OM despite elevated HK2 protein (Figure 2H,I), these data indicate that OA‐associated HK2 upregulation drives meniscal catabolism and senescence without a corresponding increase in catalytic activity.

### 3.3 Inhibition of HK2 Promotes Collagen Expression and Suppresses Senescence in an MYH10‐Dependent Manner

To further elucidate the mechanisms by which HK2 impairs meniscal ECM homeostasis and exerts pro‐senescence effects, we conducted immunoprecipitation‐mass spectrometry analysis in primary meniscal cells to identify potential HK2‐interacting proteins. Among the identified proteins, MYH10 was identified as an HK2‐interacting protein, with 54 unique peptides accounting for 46% sequence coverage of MYH10 (Figure 3A,B; Figure S3A). Previous research has indicated that the association of *COL1A1* mRNA with MYH10 is essential for coordinated COL1A1 synthesis [37] and that MYH10 plays a critical role in maintaining ECM homeostasis [38]. We subsequently confirmed the interaction between HK2 and MYH10 in primary meniscal cells (Figure 3C,D). RNA immunoprecipitation (RIP)‐RT‐qPCR assays further demonstrated that *COL1A1* mRNA was significantly enriched in MYH10 immunoprecipitates from primary meniscal cells, supporting an association between MYH10 and *COL1A1* mRNA (Figure 3E).

**FIGURE 3:**
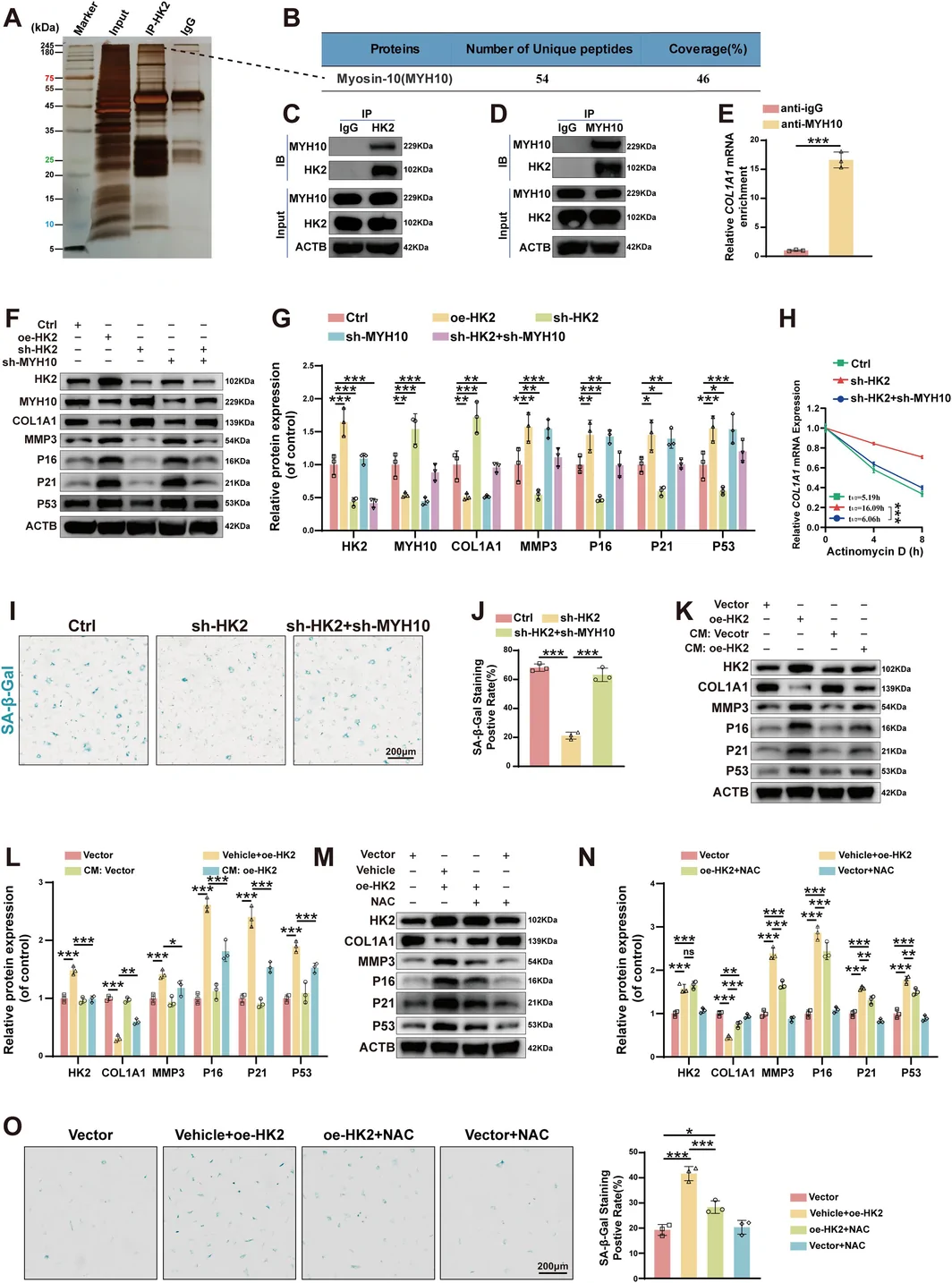
Inhibition of HK2 promotes collagen expression and suppresses senescence in an MYH10‐dependent manner. (A, B) Identification of HK2‐interacting proteins by immunoprecipitation‐mass spectrometry (IP‐MS). (C, D) Co‐IP of HK2 and MYH10 in meniscal cells (*n* = 3). (E) RIP‐RT‐qPCR showing enrichment of *COL1A1* mRNA in MYH10 immunoprecipitates in primary meniscal cells (*n* = 3). (F, G) Western blot and quantification of HK2, COL1A1, MMP3, P16, P21, P53, and ACTB with HK2 knockdown, MYH10 knockdown, or HK2 overexpression (*n* = 3). (H, I) SA‐β‐Gal staining and quantification after HK2 or MYH10 knockdown (*n* = 3). Scale bars = 200 μm. (J) *COL1A1* mRNA half‐life after HK2 or MYH10 knockdown (*n* = 3). (K, L) Western blot and quantification of HK2, COL1A1, MMP3, P16, P21, P53, and ACTB in directly transfected meniscal cells (Vector and oe‐HK2) and in normal recipient meniscal cells co‐cultured with vector‐ or HK2‐overexpressing donor cells using a Transwell system (*n* = 3). (M, N) Western blot and quantification of HK2, COL1A1, MMP3, P16, P21, P53, and ACTB in HK2‐overexpressing cells treated with or without the ROS scavenger NAC (*n* = 3). (O) Representative SA‐β‐Gal staining and quantification of HK2‐overexpressing cells treated with or without NAC (*n* = 3). Scale bars = 200 μm. Data are presented as mean ± SD, *p < 0.05, **p < 0.01, ***p < 0.001, ns, not significant.

Western blot analysis showed that both HK2 overexpression and MYH10 knockdown reduced COL1A1 expression while increasing the expression of MMP3 and senescence‐associated markers (P16, P21, and P53) (Figure 3F,G). Notably, HK2 knockdown promoted meniscal anabolic activity and suppressed catabolism and cellular senescence; however, these protective effects were reversed by MYH10 knockdown, indicating that HK2 exerts its effects in an MYH10‐dependent manner (Figure 3F,G). Consistent results were observed in SA‐β‐Gal staining, in which the anti‐senescence effects of HK2 knockdown were reversed by MYH10 knockdown (Figure 3H,I). mRNA half‐life assays demonstrated that sh‐HK2 prolonged the half‐life of *COL1A1* mRNA, whereas this effect was reversed following MYH10 knockdown (Figure 3J). Collectively, these findings indicate that HK2 suppresses meniscal ECM synthesis and promotes cellular senescence through an MYH10‐dependent, metabolism‐independent mechanism.

Having established that both ECM degradation and cellular senescence depend on MYH10, we next examined whether these two phenotypes represent independent parallel outputs or a hierarchical relationship. First, normal meniscal cells were indirectly co‐cultured with vector‐ or HK2‐overexpressing donor meniscal cells using a Transwell system. Compared with co‐culture with vector‐transfected donor cells, co‐culture with HK2‐overexpressing donor cells reduced COL1A1 and increased MMP3 and senescence‐associated markers in recipient cells (Figure 3K,L), supporting a paracrine effect of HK2‐overexpressing cells on ECM dysregulation and cellular senescence. Second, treatment of HK2‐overexpressing cells with the ROS scavenger NAC partially attenuated senescence markers, as shown by reduced P16/P21/P53 expression (Figure 3M,N) and fewer SA‐β‐Gal‐positive cells (Figure 3O), but only partially rescued COL1A1 expression (Figure 3M,N). Together, these results suggest that ECM dysregulation and cellular senescence are neither completely independent nor strictly hierarchical, but represent interconnected, partially separable downstream consequences of MYH10 loss.

### 3.4 HK2 Facilitates MYH10–PRKN Complex Formation and Promotes MYH10 Ubiquitination and Degradation

In previous experiments, we observed that HK2 overexpression reduced MYH10 protein levels (Figure 3F,G), whereas *MYH10* mRNA expression remained unchanged (Figure S3B), suggesting that HK2 regulates MYH10 at the post‐translational level. The ubiquitin‐proteasome system (UPS) is a major pathway responsible for intracellular protein degradation. To investigate whether HK2 regulates MYH10 via the UPS, co‐immunoprecipitation (Co‐IP) analysis demonstrated that HK2 overexpression increased MYH10 polyubiquitination and promoted its degradation (Figure 4A). In addition, MG132 treatment resulted in time‐dependent accumulation of MYH10 protein (Figure 4B), indicating that HK2 overexpression promotes MYH10 degradation through the UPS.

**FIGURE 4:**
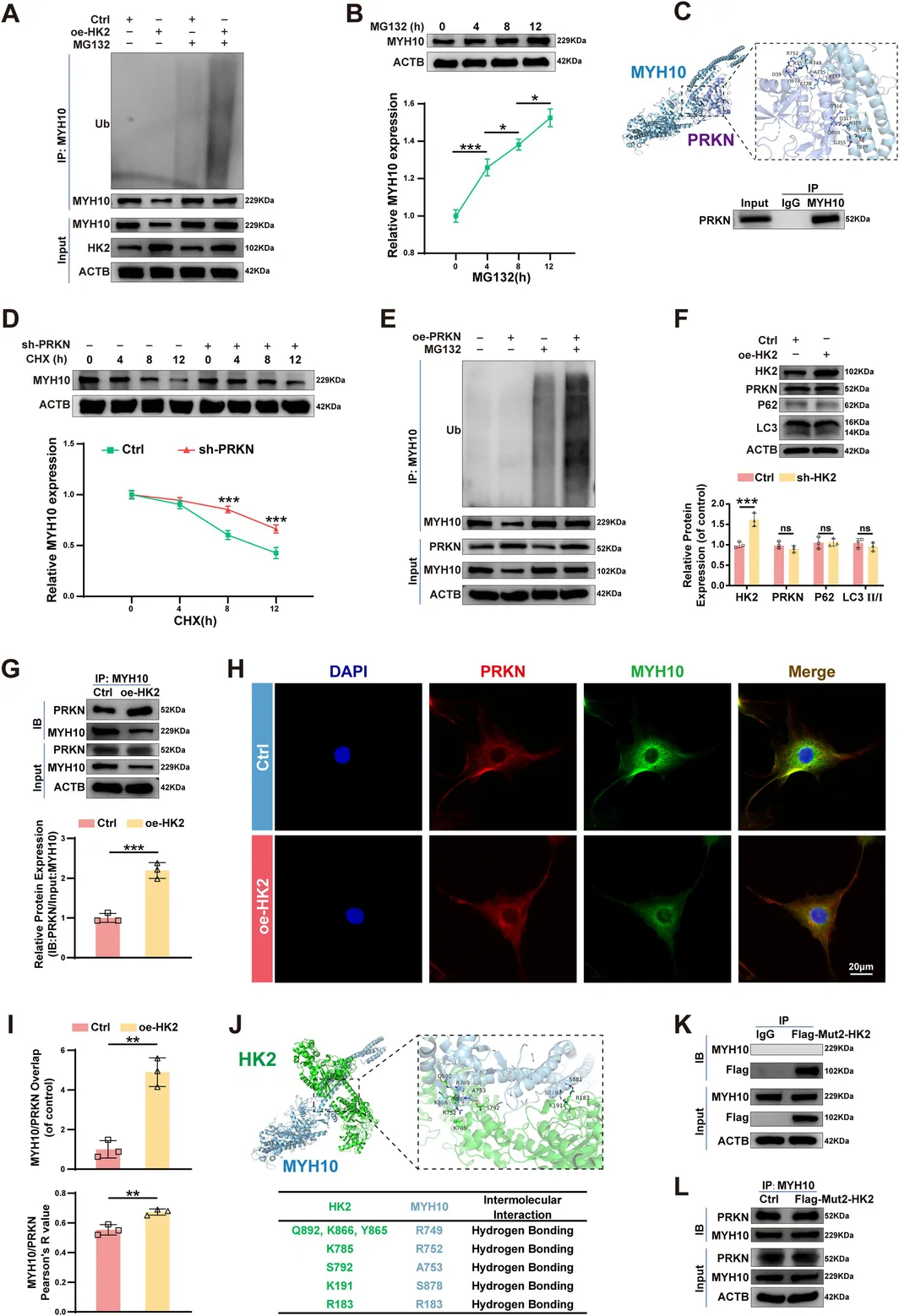
HK2 facilitates MYH10–PRKN complex formation and promotes MYH10 ubiquitination and degradation. (A) Detection of MYH10 ubiquitination levels in meniscal cells after HK2 overexpression with or without MG132 treatment (*n* = 3). (B) MYH10 protein levels in cells treated with MG132 at 0 h, 4 h, 8 h, and 12 h (*n* = 3). (C) Molecular docking analysis of MYH10–PRKN interaction and Co‐IP validation (*n* = 3). (D) MYH10 levels after PRKN knockdown (sh‐PRKN) versus control, with CHX chase (100 μg/mL) (*n* = 3). (E) MYH10 ubiquitination in PRKN‐overexpressing (oe‐PRKN) cells, with or without MG132 (*n* = 3). (F) Western blot and quantification of HK2, PRKN, P62, LC3, and ACTB after HK2 overexpression (*n* = 3). (G) Co‐IP of PRKN with MYH10 after HK2 overexpression (*n* = 3). (H, I) Immunofluorescence co‐localization of PRKN (red) and MYH10 (green) (*n* = 3). Scale bars = 20 μm. (J) Molecular docking model of the HK2–MYH10 interaction with critical interface residues. (K) Co‐IP and western blot of MYH10 in cells expressing Flag‐Mut2‐HK2 (Y865A/K866A/Q892A), a MYH10‐binding‐deficient mutant (*n* = 3). (L) Co‐IP and western blot of PRKN in cells expressing Flag‐Mut2‐HK2 (*n* = 3). Data are presented as mean ± SD, *p < 0.05, **p < 0.01, ***p < 0.001, ns, not significant.

To identify the E3 ubiquitin ligase responsible for MYH10 ubiquitination, we used the BioGRID 4.4 and UbiBrowser 2.0 databases, which predicted PRKN as a potential E3 ligase for MYH10 (Figure S3C). Molecular docking analysis suggested a potential interaction between MYH10 and PRKN, which was further confirmed using Co‐IP analysis (Figure 4C). Cycloheximide chase assays showed that PRKN knockdown delayed MYH10 degradation (Figure 4D), whereas PRKN overexpression enhanced MYH10 ubiquitination and degradation (Figure 4E), suggesting that PRKN mediates MYH10 degradation via the UPS. Importantly, HK2 overexpression did not significantly alter total PRKN, P62, or LC3 levels (Figure 4F). Together with the MG132‐sensitive accumulation of MYH10, these findings support a proteasome‐dependent route for MYH10 degradation. To further investigate the role of HK2 in MYH10–PRKN complex formation, Co‐IP analysis demonstrated that HK2 overexpression enhanced the interaction between MYH10 and PRKN (Figure 4G). IF analysis showed that HK2 overexpression reduced MYH10 expression and increased its co‐localization with PRKN (Figure 4H,I). These findings support the hypothesis that HK2 promotes MYH10 ubiquitination and degradation by facilitating MYH10–PRKN complex formation.

We next asked whether PRKN is functionally required for the downstream effects of HK2 by knocking down PRKN in HK2‐overexpressing meniscal cells. sh‐PRKN prevented HK2‐induced MYH10 downregulation, restored COL1A1 protein expression, reduced MMP3 (Figure S3D), prolonged *COL1A1* mRNA half‐life (Figure S3F), and attenuated senescence, as reflected by reduced P16/P21/P53 expression and fewer SA‐β‐Gal‐positive cells (Figure S3D,E). These results establish PRKN‐catalysed MYH10 ubiquitination as a central node in the HK2‐driven catabolic and senescent phenotype.

To determine whether the effect of HK2 on MYH10–PRKN complex formation depended on HK2–MYH10 interaction, molecular docking analysis identified seven candidate interface residues between HK2 and MYH10, including K183 and R191 within the N‐terminal hexokinase domain (residues 16–458), as well as K785, S792, Y865, K866, and Q892 within the C‐terminal hexokinase domain (residues 464–906) (Figure 4J). Notably, K183 and R191 are located within the N‐terminal catalytic cleft adjacent to the active site residue D209, and mutagenesis of these residues would likely impair its glucose phosphorylation activity. To specifically investigate whether the scaffolding function of HK2, independent of its catalytic activity, mediates MYH10 degradation, we selected three surface‐exposed residues on the C‐terminal domain (Y865, K866, and Q892) that are spatially separated from both catalytic active sites (D209 and D657) and are not involved in glucose or ATP binding. Alanine substitution of these residues (Y865A/K866A/Q892A; designated as Flag‐Mut2‐HK2) was designed to preserve enzymatic activity while disrupting protein–protein interactions. As expected, the mutant retained wild‐type hexokinase activity (Figure S3G) but showed a marked loss of association with MYH10 (Figure 4K). Consequently, this binding‐deficient mutant failed to promote MYH10–PRKN complex formation (Figure 4L), demonstrating that HK2 promotes MYH10 ubiquitination through a non‐catalytic scaffolding mechanism. Because this mutant retained enzymatic activity yet lost downstream function, these findings further confirm that the pathogenic effects of HK2 track its binding capacity rather than its catalytic activity.

### 3.5 PRKN Induces K48‐Linked Ubiquitination of MYH10, With K410 Identified as a Critical Ubiquitination Site

We next investigated the ubiquitin linkage specificity of PRKN‐mediated ubiquitination of MYH10 and found that PRKN promoted K48‐linked, but not K63‐linked, ubiquitination of MYH10 (Figure 5A,B). To identify the specific ubiquitination sites on MYH10, we analysed the PhosphoSitePlus database, which identified three lysine residues (K232, K410, and K828) supported by more than five independent studies (Figure 5C). Site‐directed mutagenesis was subsequently performed to substitute these lysine residues with arginine (K232R, K410R, and K828R), and the effects on ubiquitination were evaluated. Among the three mutants, only the K410R variant significantly attenuated MYH10 ubiquitination (Figure 5D–F). Accordingly, K410 emerged as a critical ubiquitin‐acceptor site among the candidate lysines tested. Sequence alignment analysis further demonstrated that the K410 residue is highly conserved across multiple mammalian species, including humans, mice, rats, and bovines (Figure 5G).

**FIGURE 5:**
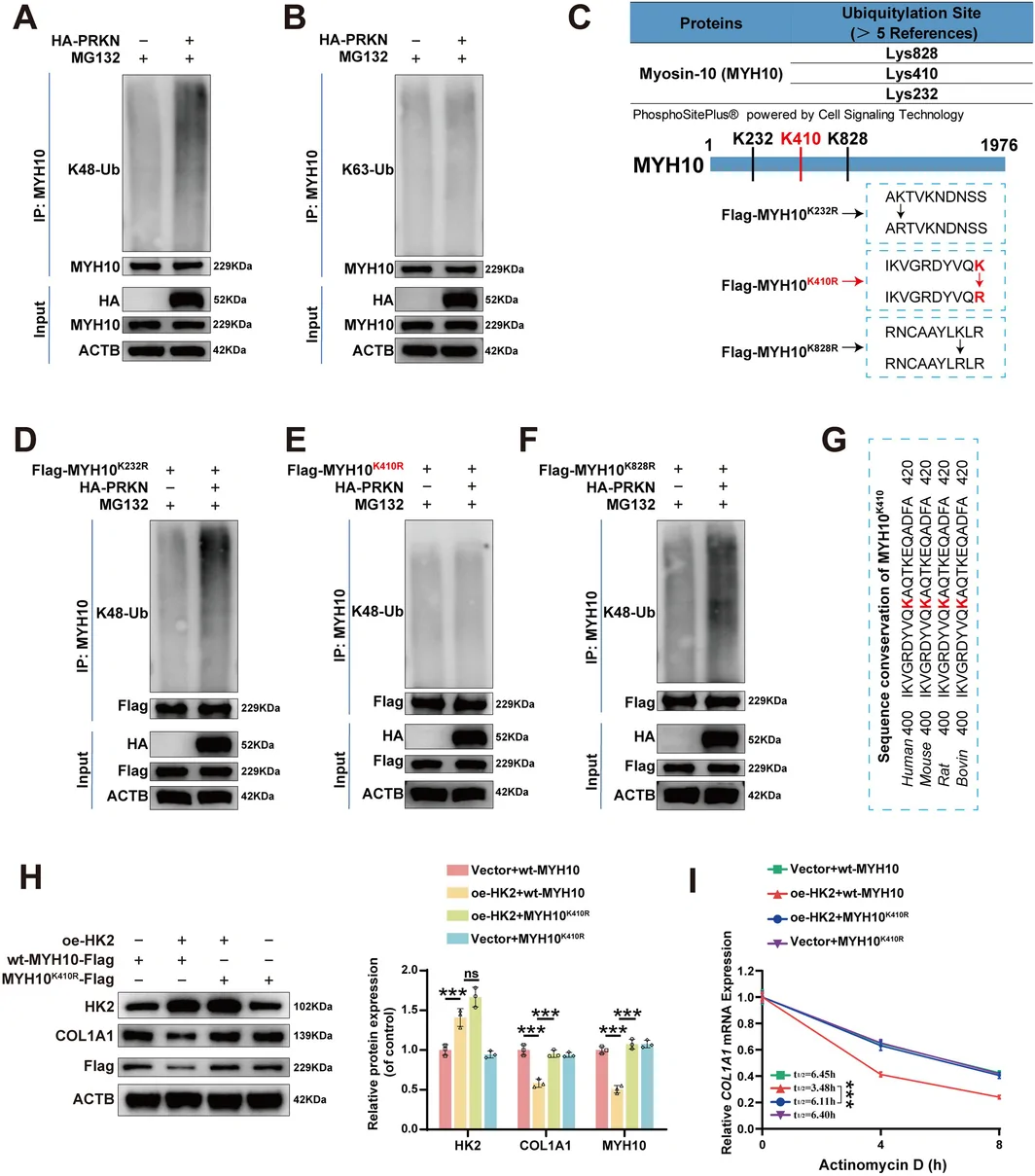
PRKN mediates K48‐linked ubiquitination of MYH10, with K410 as the critical ubiquitination site. (A, B) K48‐ and K63‐linked ubiquitination of MYH10 in HA‐PRKN‐overexpressing cells (*n* = 3). (C) MYH10 ubiquitination sites predicted by PhosphoSitePlus (> 5 references) and schematic of MYH10 point mutants (Flag‐MYH10^K232R^, Flag‐MYH10^K410R^, and Flag‐MYH10^K828R^). (D–F) Ubiquitination analysis of Flag‐MYH10^K232R^, Flag‐MYH10^K410R^, and Flag‐MYH10^K828R^ co‐expressed with HA‐PRKN by IP/IB (*n* = 3). (G) Sequence alignment showing conservation of K410 across human, mouse, rat, and bovine. (H, I) Western blot and quantification of HK2, COL1A1, and MYH10 (H) and *COL1A1* mRNA half‐life (I) in HK2‐overexpressing cells co‐expressing wt‐MYH10 or MYH10^K410R^ (*n* = 3). Data are presented as mean ± SD, ***p < 0.001, ns, not significant.

To determine whether K410 ubiquitination is functionally required for HK2‐induced MYH10 degradation and its downstream effects, we co‐expressed HK2 with wild‐type MYH10 (wt‐MYH10) or the ubiquitination‐resistant mutant MYH10^K410R^. Under HK2 overexpression, wt‐MYH10 was degraded, whereas MYH10^K410R^ remained stable; consequently, K410R preserved COL1A1 protein levels and prolonged *COL1A1* mRNA half‐life compared with wt‐MYH10 (Figure 5H,I). These data indicate that K410 ubiquitination is required for HK2‐mediated MYH10 degradation and the consequent destabilisation of *COL1A1* mRNA.

### 3.6 Non‐Metabolic Function of HK2 Aggravates OA Progression

To determine whether overexpression of catalytically inactive HK2 could aggravate OA progression in vivo, we constructed adeno‐associated viruses expressing either wild‐type HK2 (AAV‐HK2) or catalytically inactive HK2 (AAV‐mut‐HK2), which were intra‐articularly injected into the knee joints of mice with DMM‐induced OA (Figure 6A). GFP fluorescence was detected in the meniscus, articular cartilage, and synovium following intra‐articular AAV9 administration, confirming successful transduction across multiple intra‐articular tissues (Figure S3H). Following DMM surgery, vocalisation tests indicated substantial pain in the mice; however, treatment with either AAV‐HK2 or AAV‐mut‐HK2 significantly exacerbated joint pain compared with that in the DMM group (Figure 6B). Gait analysis showed that the swing/stand phase ratio was significantly increased in the DMM group, whereas both the AAV‐HK2 and AAV‐mut‐HK2 groups exhibited further deterioration in locomotor behaviour (Figure 6C). In contrast, hot plate tests revealed no significant difference in the reduced thermal pain threshold induced by DMM between the AAV‐HK2 and AAV‐mut‐HK2 groups (Figure S4E). Micro‐computed tomography (micro‐CT) imaging demonstrated prominent osteophyte formation in the DMM group, which was further aggravated by overexpression of either form of HK2 (Figure 6D). In addition, SO/FG staining of the meniscus showed that treatment with AAV‐HK2 or AAV‐mut‐HK2 further aggravated DMM‐induced meniscal degeneration (Figure 6E), accompanied by reduced Type I collagen intensity and increased numbers of MMP3‐ and P21‐positive cells (Figure 6F,G). Furthermore, SO/FG staining of cartilage tissues demonstrated that administration of AAV‐HK2 or AAV‐mut‐HK2 exacerbated proteoglycan loss and OA progression in DMM mice (Figure S4A,B). However, overexpression of either wild‐type or mutant HK2 did not further aggravate synovial inflammation in DMM‐induced OA mice (Figure S4C,D). Collectively, these findings indicated that HK2 exacerbates meniscal senescence and cartilage degeneration through its non‐metabolic function in DMM‐induced OA mice.

**FIGURE 6:**
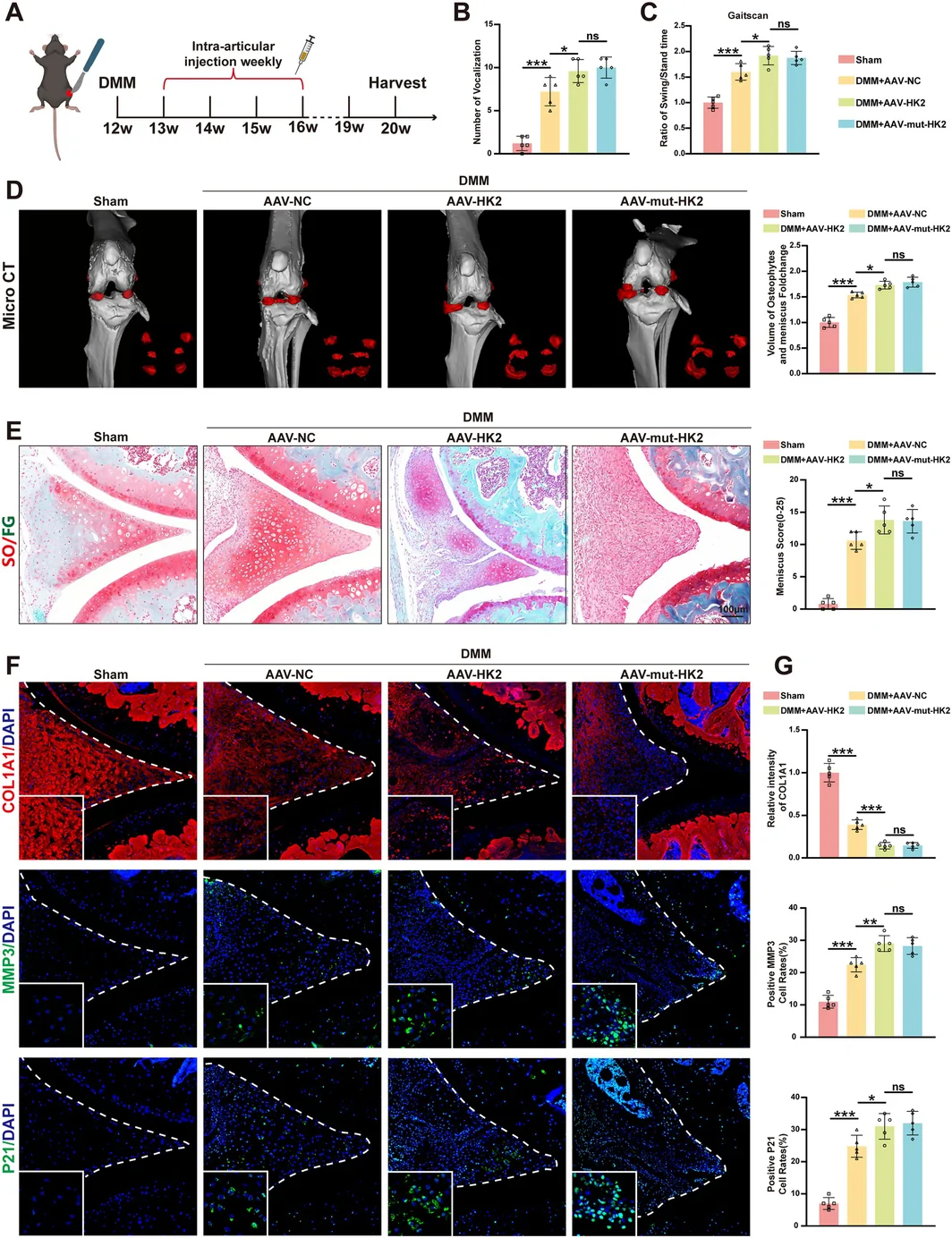
Non‐metabolic function of HK2 aggravates OA progression. (A) Schematic representation of the DMM surgery timeline and the intra‐articular injection schedule for AAV‐HK2 and AAV‐mut‐HK2. (B) Knee pain by vocalisation test (*n* = 5 mice per group). (C) Hindlimb swing/stand phase ratio determined by gait analysis (*n* = 5 mice per group). (D) 3D micro‐CT reconstructions showing osteophyte volume (red) and quantification (*n* = 5 mice per group). (E) SO/FG staining and meniscus scores of meniscal tissues. Scale bars = 100 μm (*n* = 5 mice per group). (F, G) Immunofluorescence for COL1A1 (red), MMP3 (green), and P21 (green) and quantification (*n* = 5 mice per group). Groups in B–G: Sham, DMM + AAV‐NC, DMM + AAV‐HK2, DMM + AAV‐mut‐HK2. Data are presented as mean ± SD, *p < 0.05, **p < 0.01, ***p < 0.001, ns, not significant.

### 3.7 Purpurin Inhibits MYH10 Ubiquitination by Disrupting the HK2–MYH10 Interaction

To explore potential therapeutic strategies to counter HK2‐driven MYH10 degradation in OA, we performed virtual screening of the Traditional Chinese Medicine Active Compound Library (HY‐L065, MCE) to identify small molecules that disrupt the HK2–MYH10 interaction. Virtual screening of 3219 natural products against the HK2 pocket centered on the MYH10‐interacting residues Y865/K866/Q892 yielded five lead compounds with binding energies more favourable than −5.0 kcal/mol (Figure 7A; Figure S5A–C). Among them, only two (arcyriaflavin A and purpurin) had not been previously reported in OA; both showed no cytotoxicity in meniscal cells at 15 μM, as determined by CCK‐8 assays (Figure 7B,C). Furthermore, SA‐β‐Gal staining demonstrated that purpurin significantly reduced cellular senescence in OA meniscal cells (Figure 7D,E) and was, therefore, selected for further investigation. We next examined whether purpurin could restore MYH10 expression under HK2 overexpression and found that purpurin significantly upregulated MYH10 protein in HK2‐overexpressing meniscal cells in a concentration‐dependent manner (Figure 7F). Furthermore, purpurin effectively reversed HK2 overexpression‐induced downregulation of MYH10 (Figure 7G). Co‐IP assays confirmed that purpurin reduced the interaction between MYH10 and PRKN, as well as ubiquitination‐mediated degradation of MYH10 (Figure 7H,I). To directly assess whether purpurin regulates *COL1A1* mRNA stability, we performed actinomycin D chase assays in HK2‐overexpressing cells. Purpurin treatment significantly prolonged the *COL1A1* mRNA half‐life (Figure 7J), consistent with its restoration of MYH10 protein and reduction of MYH10 ubiquitination.

**FIGURE 7:**
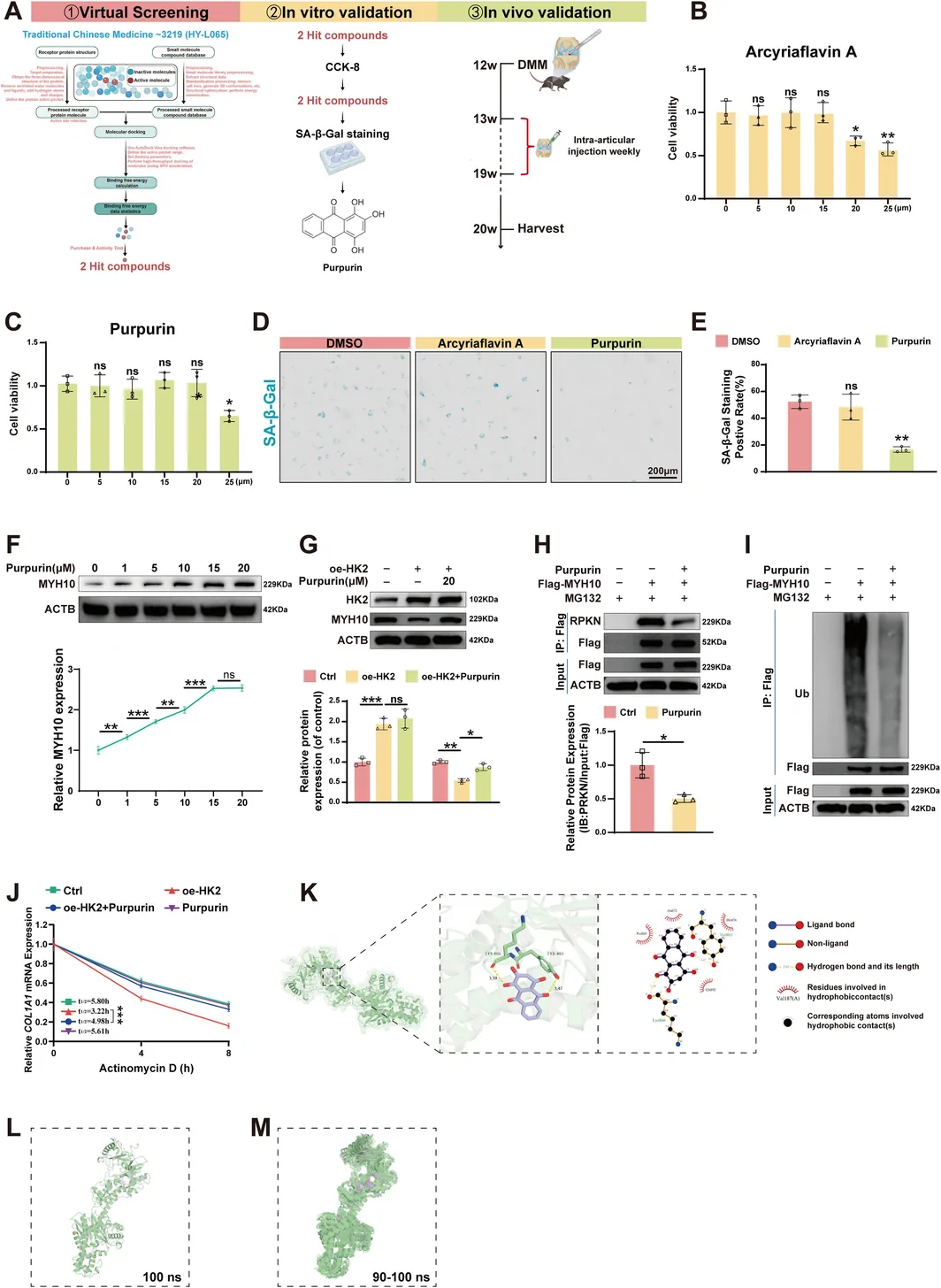
Purpurin disrupts the HK2–MYH10 interaction and inhibits MYH10 degradation. (A) Schematic of the virtual screening strategy to identify small molecules disrupting the HK2–MYH10 interaction. (B, C) Cell viability (CCK‐8) of meniscal cells treated with arcyriaflavin A or purpurin (*n* = 3). (D, E) SA‐β‐Gal staining and quantification in OA meniscal cells treated with the two compounds for 24 h (*n* = 3). Scale bars = 200 μm. (F) MYH10 levels in cells treated with increasing purpurin concentrations, with quantification (*n* = 3). (G) Western blot of HK2, MYH10, and ACTB in HK2‐overexpressing cells with or without purpurin, with quantification (*n* = 3). (H) Co‐IP showing the effect of purpurin on the MYH10–PRKN interaction in HK2‐overexpressing cells (*n* = 3). (I) MYH10 ubiquitination levels after purpurin treatment, assessed by IP (*n* = 3). (J) *COL1A1* mRNA half‐life determined by actinomycin D chase assays in HK2‐overexpressing cells treated with or without purpurin (*n* = 3). (K) Molecular docking analysis of purpurin into the MYH10‐binding pocket of HK2; the most stable conformation after 100 ns molecular dynamics (MD) simulation is shown. (L) Snapshot structure of the HK2‐purpurin complex at 100 ns of MD simulation. (M) Superposition of HK2‐purpurin complex conformations from the last 10 ns (90–100 ns) of MD simulation. Data are presented as mean ± SD, *p < 0.05, **p < 0.01, ***p < 0.001, ns, not significant.

Molecular docking showed that purpurin occupies the MYH10‐binding pocket of HK2 via hydrogen bonds with LYS‐866/TYR‐865 and hydrophobic interaction with GLN‐892 (Figure 7K). 100 ns molecular dynamics simulations confirmed the stable binding of the purpurin‐HK2 complex, as evidenced by consistent RMSD, Rg, SASA, and secondary structure evolution profiles (Figure S5D–G). The binding mechanism was further supported by sustained hydrogen bonds, low overall pocket flexibility, a single low‐energy state on the free energy landscape, and MM/GBSA analysis indicating van der Waals‐dominated binding energy (Figure S5H–O). Simulation snapshots consistently demonstrated stable retention of purpurin in the HK2 pocket (Figure 7L,M). These computational findings provide a structural rationale for purpurin‐mediated disruption of the HK2–MYH10 interaction observed in Co‐IP assays.

### 3.8 Purpurin Alleviates DMM‐Induced Meniscus Degeneration in OA Mice

To evaluate the therapeutic efficacy of purpurin in vivo, 12‐week‐old mice received weekly intra‐articular injections of purpurin or vehicle following DMM surgery. Knee joints were harvested 8 weeks postoperatively for comprehensive assessment of OA progression (Figure 8A). Vocalisation tests revealed that purpurin effectively alleviated DMM‐associated joint pain (Figure 8B), while gait analysis demonstrated that purpurin improved locomotor deficits by reducing the swing/stand phase ratio (Figure 8C). In addition, hot plate tests showed that purpurin significantly restored the reduced thermal pain threshold observed in DMM mice (Figure S6E).

**FIGURE 8:**
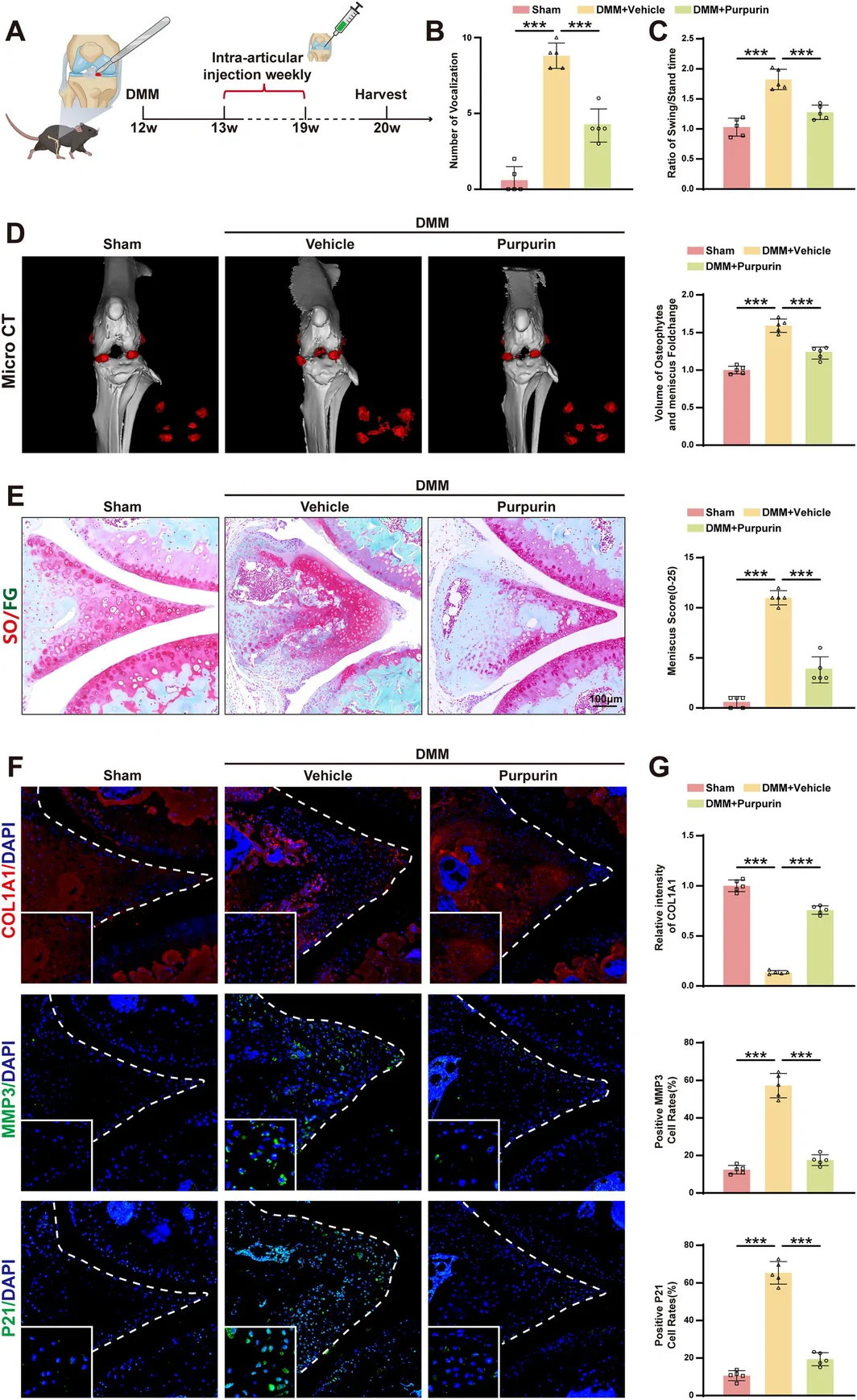
Purpurin alleviates DMM‐induced meniscus degeneration in OA mice. (A) Schematic representation of the DMM surgery timeline and the intra‐articular injection schedule for purpurin. (B) Knee pain by vocalisation test (*n* = 5 mice per group). (C) Hindlimb swing/stand phase ratio determined by gait analysis (*n* = 5 mice per group). (D) 3D micro‐CT reconstructions showing osteophyte volume (red) and quantification (*n* = 5 mice per group). (E) SO/FG staining and meniscus scores of meniscal tissues. Scale bars = 100 μm (*n* = 5 mice per group). (F, G) Immunofluorescence for COL1A1 (red), MMP3 (green), and P21 (green) and quantification (*n* = 5 mice per group). Groups in B–G: Sham, DMM + vehicle, DMM + purpurin. Data are presented as mean ± SD, ***p < 0.001.

Importantly, micro‐CT analysis revealed a substantial reduction in osteophyte formation in purpurin‐treated mice compared with control mice (Figure 8D). SO/FG staining of meniscal tissues demonstrated that purpurin attenuated DMM‐induced meniscal degeneration (Figure 8E). IF staining further showed that purpurin partially restored COL1A1 expression while reducing MMP3 and P21 expression (Figure 8F,G). Moreover, purpurin significantly reduced proteoglycan loss in cartilage tissues (Figure S6A,B) and alleviated synovial inflammation in DMM‐induced OA mice (Figure S6C,D). Collectively, these findings demonstrate that purpurin effectively attenuates OA progression in vivo, consistent with its in vitro disruption of the pathogenic HK2–MYH10 interaction.

## 4 Discussion

In OA, chondrocytes undergo a metabolic shift towards glycolysis, which impairs mitochondrial membrane potential, disrupts mitochondrial morphology, and promotes ECM degradation [39, 40, 41]. Inhibition of HK2 in OA chondrocytes suppressed catabolic pathways and attenuated inflammatory responses, whereas HK2 overexpression stimulated inflammatory and catabolic activity [40]. Similarly, energy metabolism in OM shifts from mitochondrial oxidative phosphorylation towards glycolysis [10]. In the present study, we observed a significant upregulation of HK2 in OM compared with that in NM. Mechanistically, our data support a model in which HK2 promotes PRKN‐mediated MYH10 degradation, with MYH10 depletion contributing to two major downstream pathological outcomes. First, reduced MYH10 destabilised *COL1A1* mRNA, thereby suppressing meniscal anabolic activity and promoting ECM catabolism. Second, HK2 overexpression induced mitochondrial dysfunction, reduced mitochondrial membrane potential, and increased ROS generation, thereby promoting cellular senescence; the MYH10‐dependent rescue experiments further indicate that MYH10 is required for the full pro‐senescence effect of HK2. Notably, these regulatory effects were independent of the metabolic activity of HK2, as supported by four converging lines of evidence: unchanged total hexokinase activity in OM despite elevated HK2 protein (Figure 2H,I); retention of catabolic and pro‐senescence effects by the catalytically inactive mutant (D209A/D657A) (Figure 2J,K); similar exacerbation of OA by AAV‐mut‐HK2 and AAV‐HK2 in vivo (Figure 6); and loss of function by the catalytically intact but binding‐deficient mutant (Y865A/K866A/Q892A) (Figure 4K,L). Our data further indicate that HK2‐induced ECM dysregulation and cellular senescence are both MYH10‐dependent but are not strictly hierarchical. Transwell co‐culture with HK2‐overexpressing donor cells induced ECM dysregulation and senescence‐associated changes in normal recipient meniscal cells, supporting a paracrine contribution of HK2‐overexpressing cells to both phenotypes (Figure 3K,L), whereas ROS scavenging with NAC attenuated senescence‐associated markers, including reduced P16/P21/P53 expression and SA‐β‐Gal positivity, but only partially restored COL1A1 expression (Figure 3M–O). Together, these findings suggest that ECM dysregulation and cellular senescence are interconnected but partially separable downstream consequences of HK2–MYH10 dysregulation, with paracrine signalling providing an additional mechanism through which HK2‐overexpressing cells can influence neighbouring meniscal cells.

Notably, although HK2 protein was elevated in the OA meniscus, total hexokinase activity remained unchanged. This dissociation between protein abundance and measurable enzyme activity may reflect several non‐mutually exclusive mechanisms: (i) post‐translational modifications that regulate catalytic activity independently of protein abundance—for example, SUMOylated HK2 inhibits glucose consumption and lactate production, whereas phosphorylation of HK2 enhances its catalytic activity [26, 42]; (ii) product feedback and allosteric inhibition of HK2 by accumulated metabolites, such as glucose‐6‐phosphate, in degenerating tissue [39, 43]; (iii) engagement of a fraction of HK2 in non‐catalytic conformations or in protein complexes as a scaffold [44]; and (iv) changes in the subcellular distribution of HK2 that affect its catalytic availability in situ—for example, nuclear HK2 regulates cell stemness independently of its enzymatic activity [25]. These possibilities remain to be examined in future studies.

Non‐metabolic functions of glycolytic enzymes are increasingly recognised across diverse diseases [25, 26, 27, 35, 45]. Previously reported non‐metabolic roles of HK2 include nuclear regulation of stem/progenitor function [25], SUMOylation‐governed mitochondrial binding in prostate cancer [26], protein‐kinase activity towards SMPD1 [27], and scaffolding of the CD133–USP11 interaction, which reduces CD133 ubiquitination and degradation [35]. Notably, that previously reported scaffold function promotes the stabilisation of an interacting protein, whereas our study reveals the opposite outcome in the OA meniscus: HK2 binding to MYH10 facilitates MYH10–PRKN complex formation, promoting K48‐linked ubiquitination of MYH10 at K410 and its subsequent proteasomal degradation, thereby destabilising *COL1A1* mRNA and driving meniscal senescence and OA progression. K410 is located within the N‐terminal myosin motor/head domain of MYH10, and our mutagenesis data identify K410 as a critical ubiquitin‐acceptor residue for PRKN‐mediated MYH10 ubiquitination. Although PRKN is classically involved in PINK1‐dependent mitochondrial quality control, this function is not mutually exclusive with the MYH10 proteasomal pathway identified here. These findings suggest that HK2 can promote the degradation of an interacting partner through an E3 ligase–substrate scaffolding mechanism, and reveal a previously unrecognised link between the non‐metabolic function of HK2 and meniscal degeneration/OA. Moreover, other glycolytic enzymes, including phosphoglycerate kinase 1 and pyruvate kinase M2, possess non‐classical functions as protein kinases that promote tumour progression by phosphorylating multiple protein substrates [45, 46]. Together with previous studies, our findings expand the current understanding of the non‐classical functions of glycolysis‐related enzymes.

In OA, the severity of meniscal tears correlates with reduced Type I collagen levels, while increased collagen expression is linked to tissue repair [8, 10, 11, 47, 48, 49, 50, 51, 52]. Traumatic meniscal tears similarly feature a loss of Type I collagen [53]. Together, these observations establish that Type I collagen integrity is essential for meniscal homeostasis. Accordingly, diverse reparative strategies—including growth factors, epigenetic regulators, and stem cell‐based therapies—converge on the restoration of Type I collagen expression to promote meniscal healing [8, 10, 49, 51]. Thus, maintaining collagen homeostasis is a central objective in meniscal repair. Given that collagen destruction is a hallmark of meniscal degeneration, our finding that HK2 is upregulated in the OM and drives collagen loss may partially account for the compromised ECM integrity observed during OA progression.

The findings of the present study demonstrate that HK2‐mediated regulation of meniscal cells is dependent on MYH10. Although our RIP‐RT‐qPCR data demonstrate an association between MYH10 and *COL1A1* mRNA, they do not establish direct protein–RNA binding. Previous work suggests that nonmuscle myosin IIB can associate with collagen mRNAs through the RNA‐binding protein LARP6; whether a similar intermediary complex operates in meniscal cells remains to be determined [37]. A previous study has shown that MYH10 deficiency during alveolar formation leads to ECM abnormalities characterised by reduced collagen expression and increased matrix metalloproteinase activity, which are closely associated with the pathogenesis of emphysema [38]. In addition, MYH10 knockdown suppresses proliferation of serous ovarian cancer cells in both in vivo and in vitro settings [54]. Further in vivo experiments in the present study demonstrated that intra‐articular injection of AAV‐mut‐HK2 into DMM‐induced OA mice exacerbated Type I collagen loss and increased expression of catabolic enzymes in the meniscus, while simultaneously promoting meniscal senescence and accelerating OA progression. These findings suggest that HK2 upregulation disrupts meniscal ECM homeostasis and promotes meniscal senescence, thereby accelerating OA progression. Collectively, these results identify HK2 as a potential therapeutic target for meniscal degeneration.

Purpurin, a natural anthraquinone, has previously been reported to exert protective effects against rheumatoid arthritis. In adjuvant‐induced arthritic rats, purpurin attenuates joint inflammation by downregulating IL‐6, TNF‐α, IL‐1β, and MMP3, upregulating IL‐10, and restoring FOXP3‐mediated CD4+/CD8+ T‐cell homeostasis [55]. In addition, purpurin exhibits anti‐aging properties, as demonstrated by its ability to ameliorate D‐galactose‐induced memory impairment, hippocampal microglial activation, and oxidative stress in mice [56, 57]. These findings suggest that purpurin possesses both anti‐arthritic and anti‐aging pharmacological activities. In the present study, using virtual screening combined with in vivo and in vitro validation experiments, we demonstrated that purpurin disrupted the HK2–MYH10 interaction in vitro, accompanied by reduced MYH10 ubiquitination, restoration of MYH10, reduced expression of senescence‐associated markers, and restoration of collagen expression. Ultimately, purpurin mitigated OA progression in DMM‐induced OA mice. These findings reveal a previously unrecognised anti‐meniscal senescence property of purpurin and highlight its potential as a lead compound for DMOAD development. Unlike direct inhibition of HK2 enzymatic activity, which may disrupt systemic glucose homeostasis, purpurin may enable more precise pathological intervention by disrupting the non‐metabolic HK2–MYH10 interaction and downstream MYH10‐mediated signalling while potentially avoiding direct inhibition of HK2 catalytic activity.

The HK2–MYH10 axis may also influence the endogenous repair capacity of the meniscus. Meniscus progenitor/stem cells [12] are thought to contribute to tissue homeostasis and injury repair. Although the role of MYH10 has not been directly examined in meniscus progenitor cells, MYH10‐encoded non‐muscle myosin IIB has been shown to regulate stem/progenitor cell behaviours relevant to regeneration, including asymmetric division and polarised motility [58]. Therefore, HK2‐mediated degradation of MYH10, together with the induction of cellular senescence, may impair the regenerative capacity of meniscus progenitor cells, whereas restoration of MYH10 by purpurin may help preserve this regenerative niche. Future studies are warranted to directly determine whether the HK2–MYH10 axis regulates meniscus progenitor cell function and endogenous meniscal repair.

This study has several limitations that should be acknowledged. First, a meniscal specimen bank covering different OA stages was unavailable, limiting a comprehensive correlation analysis between HK2 expression and OA severity. Second, the control and OA donors were not age‐matched, because normal menisci are obtainable only from relatively young amputation patients; as age itself affects cellular senescence, demographic confounding cannot be fully excluded. Moreover, TBHP models oxidative stress‐associated senescence‐like changes rather than natural replicative senescence, which should be considered when interpreting our in vitro findings. Third, mitophagy‐specific markers and mitophagy flux were not directly assessed; therefore, a concurrent contribution of the canonical PINK1–PRKN mitochondrial quality‐control pathway to the observed mitochondrial alterations cannot be excluded. Fourth, although our findings indicated that inhibiting ubiquitination‐mediated MYH10 degradation could preserve meniscal ECM characteristics, detailed biomechanical analyses were not conducted. Fifth, although purpurin disrupts the HK2–MYH10 interaction in vitro, its translational potential remains to be established: its pharmacokinetics in joint tissues (half‐life, residence time and tissue distribution), systemic safety, and long‐term efficacy have not been evaluated, and no pharmacokinetic data are available to confirm that effective concentrations are maintained throughout the 7‐day dosing interval. In addition, given the documented antioxidant and anti‐inflammatory activities of purpurin, off‐target contributions to its in vivo efficacy cannot be excluded, and the causal relationship between disruption of the HK2–MYH10 interaction and the observed therapeutic effects has not been formally established. The present findings therefore support purpurin as a lead compound for DMOAD development rather than a clinical candidate, and dedicated pharmacokinetic/pharmacodynamic, tissue‐distribution, and toxicology studies are warranted before clinical translation can be considered. Finally, all in vivo experiments were performed exclusively in 12‐week‐old male C57BL/6 mice; given the higher OA prevalence in women and the potential protective role of oestrogen, these findings cannot be directly extrapolated to females, and future studies including female mice are warranted.

In summary, our findings demonstrate that HK2 expression is upregulated in the OM, providing insights into the pathological role of HK2 in OA progression. Elevated HK2 levels promote interaction between MYH10 and the E3 ubiquitin ligase PRKN, resulting in MYH10 ubiquitination and degradation. Reduced MYH10 levels subsequently impair its association with *COL1A1* mRNA, thereby shortening the mRNA half‐life and disrupting meniscal ECM homeostasis while promoting cellular senescence. Collectively, dysregulation of the HK2/MYH10/PRKN axis compromises meniscal ECM integrity and accelerates meniscal senescence and OA progression.

## Author Contributions

**Zhencan Lin:** conceptualisation, formal analysis, investigation, project administration, writing – original draft. **Ming Li:** data curation, methodology, project administration, writing – original draft. **Wei Li:** data curation, formal analysis, project administration, validation. **Tianfa Xu:** data curation, formal analysis, software. **Zongrui Jiang:** software, visualisation. **Yanlin Zhong:** validation, visualisation. **Aishan He:** supervision. **Xiaoyi Zhao:** conceptualization, funding acquisition, project administration, writing – review and editing. **Dianbo Long:** conceptualisation, resources, supervision, writing – review and editing. **Yan Kang:** conceptualisation, funding acquisition, resources, supervision, writing – review and editing.

## Funding

This study was supported by the National Natural Science Foundation of China (82604762). The Basic and Applied Basic Research Foundation of Guangdong Province (2025A1515012420, 2026A1515012724, 2024A1515030086).

## Ethics Statement

This study was approved by the Ethics Committee of the First Affiliated Hospital of Sun Yat‐sen University (approval no. 2021‐334). All procedures were performed in accordance with the ethical standards of this committee. All animal experiments were approved by the Committee of Animal Care and Use of Sun Yat‐sen University (SYSU‐IACUC‐2024001532).

## Consent

All authors have read and approved the manuscript and agreed to submit it to the journal.

## Conflicts of Interest

The authors declare no conflicts of interest.

## Supporting information


**Figure S1:** HK2 is upregulated in OA meniscus, DMM mouse model, and naturally aged mouse model. (A) Quantitative analysis of SA‐β‐Gal staining in normal and OA meniscal cells (*n* = 3). (B, C) Western blot and quantification of HK2, COL1A1, MMP3, P16, P21, P53, and ACTB in normal and OA meniscus tissues (*n* = 5). (D) *HK2* mRNA expression by RT‐qPCR (*n* = 6). (E) HK2 protein expression in TBHP‐induced senescence‐like meniscal cells (*n* = 3). (F) *HK2* mRNA expression in TBHP‐induced senescence‐like model of meniscal cells (*n* = 6). (G) Meniscus histopathological score of normal and OA meniscus (*n* = 5). (H–K) Quantification of COL1A1, MMP3, P21, and HK2 immunofluorescence (*n* = 3). (L) Immunofluorescence for HK2 and P21 in menisci of Sham, DMM, young (3 months), and aged (18 months) mice, with quantification (*n* = 3). Scale bars = 100 μm (Left) and 20 μm (Right). NM, normal meniscus; OM, OA meniscus. Data are presented as mean ± SD, *p < 0.05, **p < 0.01, ***p < 0.001.
**Figure S2:** HK2 overexpression exacerbates meniscal collagen loss and induces cellular senescence. (A, B) Quantification of P21 and ROS after HK2 overexpression (*n* = 3). (C) Schematic of catalytically inactive mut‐HK2 mutation sites. (D) Quantification of HK2, COL1A1, MMP3, P16, P21, P53, and ACTB in oe‐HK2 and mut‐HK2 cells (*n* = 3). Data are presented as mean ± SD, *p < 0.05, ***p < 0.001.
**Figure S3:** IP‐MS identification of MYH10 as an HK2‐associated protein. (A) Representative mass spectrum of an MYH10‐derived peptide. (B) MYH10 mRNA levels after HK2 overexpression (*n* = 5). (C) Venn diagram of MYH10 E3 ligases predicted by BioGRID 4.4 and UbiBrowser 2.0. (D) Western blot and quantification of HK2, COL1A1, MYH10, MMP3, P16, P21, P53, PRKN, and ACTB in HK2‐overexpressing cells with or without PRKN knockdown (sh‐PRKN) (*n* = 3). (E) SA‐β‐Gal staining and quantification of HK2‐overexpressing cells with or without sh‐PRKN (*n* = 3). Scale bars = 200 μm. (F) COL1A1 mRNA half‐life determined by actinomycin D chase assays in HK2‐overexpressing cells with or without sh‐PRKN (*n* = 3). (G) Hexokinase activity in cells overexpressing oe‐HK2 or the MYH10‐binding‐deficient mutant HK2 (Mut2‐HK2, Y865A/K866A/Q892A) (*n* = 3). (H) Representative GFP fluorescence images showing AAV9‐mediated transgene expression in the meniscus, articular cartilage, and synovium. Scale bars = 100 μm and 20 μm. Data are presented as mean ± SD, ***p < 0.001, ns, not significant.
**Figure S4:** Non‐metabolic function of HK2 aggravates OA progression. (A, B) SO/FG staining and OARSI score (*n* = 5 mice per group). Scale bars = 200 μm. (C, D) H&E‐stained synovial tissue and synovitis score (*n* = 5 mice per group). Scale bars = 200 μm (Top) and 20 μm (Bottom). (E) Hot plate test results for pain sensitivity (*n* = 5 mice per group). Groups: Sham, DMM + AAV‐NC, DMM + AAV‐HK2, DMM + AAV‐mut‐HK2. Data are presented as mean ± SD, *p < 0.05, **p < 0.01, ***p < 0.001, ns, not significant.
**Figure S5:** Virtual screening and molecular dynamics characterisation of candidate HK2–MYH10 interaction inhibitors. (A) Binding energies of the top 5 hits (all more favourable than −5.0 kcal/mol). (B, C) Docking models of arcyriaflavin A and purpurin in the MYH10‐binding pocket of HK2. (D) Time evolution of RMSD (Root Mean Square Deviation) for the protein (red) and the ligand (black). (E) Time evolution of the radius of gyration (Rg) for the protein (red), indicating the overall structural compactness. (F) Time evolution of the SASA (Solvent Accessible Surface Area) of the protein. (G) Time evolution of the number of residues in each secondary structure type (calculated by DSSP). (H) RMSF (Root Mean Square Fluctuation) of the protein amino acid residues. (I) Time evolution of the number of hydrogen bonds (H‐bonds) formed between the protein and the ligand. (J) Two‐dimensional Free Energy Landscape (FEL) of the protein‐ligand complex projected onto RMSD and radius of gyration (Rg) reaction coordinates. (K) Three‐dimensional representation of the FEL. (L) Energy convergence profile of the molecular dynamics simulation. (M, N) Decomposition analysis of MM/GBSA binding energy using the Molecular Mechanics/Generalized Born Surface Area approach. (O) Per‐residue energy decomposition of key amino acids at the binding interface.
**Figure S6:** Purpurin alleviates OA progression and synovitis in mice. (A, B) SO/FG staining and OARSI score (*n* = 5 mice per group). Scale bars = 200 μm. (C, D) H&E‐stained synovial tissue and synovitis score (*n* = 5 mice per group). Scale bars = 200 μm (top) and 20 μm (bottom). (E) Hot plate test results for pain sensitivity (*n* = 5 mice per group). Groups: Sham, DMM + Vehicle, DMM + purpurin. Data are presented as mean ± SD, *p < 0.05, ***p < 0.001.

**Table S1:** Baseline characteristics of patients (*n* = 5 per group).

**Table S2:** Primers and sequences used in this study.

**Table S3:** Antibodies used in this study.

## Data Availability

The data that support the findings of this study are available from the corresponding author upon reasonable request.
