# Supplementary material for: Purpurin Alleviates Meniscal Senescence and Osteoarthritis by Blocking the Non‐Metabolic Function of Hexokinase 2
Source: Cell Prolif. 2026 Oct 29:e70294. Online ahead of print. doi: 10.1111/cpr.70294 (PMC13719234; doi:10.1111/cpr.70294)
Supplement: Supplementary file 1 [file CPR-9999-e70294-s001.docx]

**Supplementary Table S3.** Antibodies used in this study.

| **Primary antibodies** | **Article number and manufacturer** | **Dilution for WB** | **Dilution for IF** |
| --- | --- | --- | --- |
| HK2 | 22029-1-AP, Proteintech | 1:1000 | 1:500 |
| COL1A1 | 67288-1-Ig, Proteintech | 1:5000 | - |
|  | GB11022, Servicebio | - | 1:300 |
| MMP3 | 66338-1-Ig, Proteintech | 1:5000 | - |
|  | GB11131, Servicebio | - | 1:500 |
| p16 | 10883-1-AP, Proteintech | 1:1000 | - |
| p21 | 10355-1-AP, Proteintech | 1:1000 | 1:500 |
| p53 | GB15627, Servicebio | 1:1000 | - |
| ACTB | 66009-1-Ig, Proteintech | 1:20000 | - |
| MYH10 | 19673-1-AP, Proteintech | 1:20000 | 1:300 |
| Flag tag | 20543-1-AP, Proteintech | 1:20000 | - |
| PRKN | 140601-1-AP, Proteintech | 1:1000 | 1:300 |
| SQSTM1-p62 | GB11531, Servicebio | 1:500 | - |
| LC3-I/II | 43566, Cell Signaling Technology | 1:1000 | - |
| HA Tag | MA103-25T, ABMgic | 1:20000 | - |
| Ubiquitin | 80992-1-RR, Proteintech | 1:10000 | - |
| K48-linkage Specific Polyubiquitin | 30339-1, Signalway Antibody | 1:1000 | - |
| K63-linkage Specific Polyubiquitin | 30340-1, Signalway Antibody | 1:1000 | - |
|  |  |  |  |
