# Supplementary material for: Purpurin Alleviates Meniscal Senescence and Osteoarthritis by Blocking the Non‐Metabolic Function of Hexokinase 2
Source: Cell Prolif. 2026 Oct 29:e70294. Online ahead of print. doi: 10.1111/cpr.70294 (PMC13719234; doi:10.1111/cpr.70294)
Supplement: Supplementary file 2 [file CPR-9999-e70294-s002.docx]

**Supplementary Table S2.** Primers and sequences used in this study.

| **Gene** | **Primer sequence (5’-3’)** |
| --- | --- |
| *hsa-ACTB-F* | *GAGCACAGAGCCTCGCC* |
| *hsa-ACTB-R* | *ATCACGCCCTGGTGCC* |
| *hsa-HK2-F* | *TTGACCAGGAGATTGACATGGG* |
| *hsa-HK2-R* | *CAACCGCATCAGGACCTCA* |
| *hsa-MYH10-F* | *GTAACTGGCTATATCGTTGGGG* |
| *hsa-MYH10-R* | *CTTTTGCTTGACGAACAGCAC* |
| *hsa-COL1A1-F* | *GAGGGCCAAGACGAAGACATC* |
| *hsa-COL1A1-R* | *CAGATCACGTCATCGCACAAC* |
