# Supplementary material for: Purpurin Alleviates Meniscal Senescence and Osteoarthritis by Blocking the Non‐Metabolic Function of Hexokinase 2
Source: Cell Prolif. 2026 Oct 29:e70294. Online ahead of print. doi: 10.1111/cpr.70294 (PMC13719234; doi:10.1111/cpr.70294)
Supplement: Supplementary file 3 [file CPR-9999-e70294-s003.docx]

**Supplementary Table S1.** Baseline characteristics of patients (n = 5 per group).

| **Variables** | **Normal meniscus (%)** | **Osteoarthritis meniscus (%)** |
| --- | --- | --- |
| **Age** |  |  |
| <65 | 5 (100) | 4 (80) |
| ≥65 | 0 (0) | 1 (20) |
| **Sex** |  |  |
| Male | 4 (80) | 2 (40) |
| Female | 1 (20) | 3 (60) |
| **Body Mass Index** |  |  |
| <18.5 | 0 (0) | 0 (0) |
| ≥18.5, <24 | 1 (20) | 0 (0) |
| ≥24, <28 | 3 (60) | 3 (60) |
| ≥28 | 1 (20) | 2 (40) |
| **Affected side** |  |  |
| Left | 2 (40) | 1 (20) |
| Right | 3 (60) | 4 (80) |
| **Disease duration (years)** |  |  |
| <10 | - | 3 (60) |
| ≥10 | - | 2 (40) |
| **Kellgren–Lawrence grade** |  |  |
| 0 | 5 (100) | 0 (0) |
| I | 0 (0) | 0 (0) |
| II | 0 (0) | 0 (0) |
| III | 0 (0) | 1 (20) |
| IV | 0 (0) | 4 (80) |
