# Supplementary material for: Purpurin Alleviates Meniscal Senescence and Osteoarthritis by Blocking the Non‐Metabolic Function of Hexokinase 2
Source: Cell Prolif. 2026 Oct 29:e70294. Online ahead of print. doi: 10.1111/cpr.70294 (PMC13719234; doi:10.1111/cpr.70294)
Supplement: Supplementary file 4 [file CPR-9999-e70294-s004.docx]

**Fig. S1. HK2 is upregulated in OA meniscus, DMM mouse model, and naturally aged mouse model. (A) Quantitative analysis of SA-β-Gal staining in normal and OA meniscal cells (n = 3). (B, C) Western blot and quantification of HK2, COL1A1, MMP3, P16, P21, P53, and ACTB in normal and OA meniscus tissues (n = 5). (D) *HK2* mRNA expression by RT-qPCR (n = 6). (E) HK2 protein expression in TBHP-induced senescence-like meniscal cells (n = 3). (F) *HK2* mRNA expression in TBHP-induced senescence-like model of meniscal cells (n = 6). (G) Meniscus histopathological score of normal and OA meniscus (n = 5). (H-K) Quantification of COL1A1, MMP3, P21, and HK2 immunofluorescence (n = 3). (L) Immunofluorescence for HK2 and P21 in menisci of Sham, DMM, young (3 months), and aged (18 months) mice, with quantification (n = 3). Scale bars = 100** µ**m (Left) and 20** µ**m (Right). NM, normal meniscus; OM, OA meniscus. Data are presented as mean ± SD, ^*^ *P* < 0.05, ^**^ *P* < 0.01, ^***^ *P* < 0.001.**

**Fig. S2. HK2 overexpression exacerbates meniscal collagen loss and induces cellular senescence.** (A**,** B) Quantification of P21 and ROS after HK2 overexpression (n = 3). (C) Schematic of catalytically inactive mut-HK2 mutation sites. (D) Quantification of HK2, COL1A1, MMP3, P16, P21, P53, and ACTB in oe-HK2 and mut-HK2 cells (n = 3). **Data are presented as mean ± SD**, **^*^ *P* < 0.05, ^***^ *P* < 0.001.**

**Fig. S3. IP-MS identification of MYH10 as an HK2-associated protein. (A) Representative mass spectrum of an MYH10-derived peptide. (B) *MYH10* mRNA levels after HK2 overexpression (n = 5). (C) Venn diagram of MYH10 E3 ligases predicted by BioGRID 4.4 and UbiBrowser 2.0. (D) Western blot and quantification of HK2, COL1A1, MYH10, MMP3, P16, P21, P53, PRKN, and ACTB in HK2-overexpressing cells with or without PRKN knockdown (sh-PRKN) (n = 3). (E) SA-β-Gal staining and quantification of HK2-overexpressing cells with or without sh-PRKN (n = 3). Scale bars = 200 µm. (F) *COL1A1* mRNA half-life determined by actinomycin D chase assays in HK2-overexpressing cells with or without sh-PRKN (n = 3). (G) Hexokinase activity in cells overexpressing oe-HK2 or the MYH10-binding-deficient mutant HK2 (Mut2-HK2, Y865A/K866A/Q892A) (n = 3). (H)** Representative GFP fluorescence images showing AAV9-mediated transgene expression in the meniscus, articular cartilage, and synovium. **Scale bars = 100 µm and 20 µm.** Da**ta are presented as mean ± SD, ^***^ *P* < 0.001, ns, not significant.**

**Fig. S4. Non-metabolic function of HK2 aggravates OA progression. (A, B) SO/FG staining and OARSI score (n = 5 mice per group). Scale bars = 200** µ**m. (C, D) H&E-stained synovial tissue and synovitis score (n = 5 mice per group). Scale bars = 200** µ**m (Top) and 20** µ**m (Bottom). (E) Hot plate test results for pain sensitivity (n = 5 mice per group). Groups: Sham, DMM + AAV-NC, DMM + AAV-HK2, DMM + AAV-mut-HK2. Data are presented as mean ± SD, ^*^ *P* < 0.05, ^**^ *P* < 0.01, ^***^ *P* < 0.001, ns, not significant.**

**Fig. S5. Virtual screening and molecular dynamics characterization of candidate HK2–MYH10 interaction inhibitors. (A) Binding energies of the top 5 hits (all more favorable than −5.0 kcal/mol). (B, C) Docking models of arcyriaflavin A and purpurin in the MYH10-binding pocket of HK2. (D) Time evolution of RMSD (Root Mean Square Deviation) for the protein (red) and the ligand (black). (E) Time evolution of the radius of gyration (Rg) for the protein (red), indicating the overall structural compactness. (F) Time evolution of the SASA (Solvent Accessible Surface Area) of the protein. (G) Time evolution of the number of residues in each secondary structure type (calculated by DSSP). (H) RMSF (Root Mean Square Fluctuation) of the protein amino acid residues. (I) Time evolution of the number of hydrogen bonds (H-bonds) formed between the protein and the ligand. (J) Two-dimensional Free Energy Landscape (FEL) of the protein-ligand complex projected onto RMSD and radius of gyration (Rg) reaction coordinates. (K) Three-dimensional representation of the FEL. (L) Energy convergence profile of the molecular dynamics simulation. (M, N) Decomposition analysis of MM/GBSA binding energy using the Molecular Mechanics/Generalized Born Surface Area approach. (O) Per-residue energy decomposition of key amino acids at the binding interface.**

**Fig. S6. Purpurin alleviates OA progression and synovitis in mice. (A, B) SO/FG staining and OARSI score (n = 5 mice per group). Scale bars = 200** µ**m. (C, D) H&E-stained synovial tissue and synovitis score (n = 5 mice per group). Scale bars = 200** µ**m (Top) and 20** µ**m (Bottom). (E) Hot plate test results for pain sensitivity (n = 5 mice per group). Groups: Sham, DMM + Vehicle, DMM + purpurin. Data are presented as mean ± SD, ^*^ *P* < 0.05, ^***^ *P* < 0.001.**
